# Identification and characterization of an efficient acyl-CoA: diacylglycerol acyltransferase 1 (*DGAT1*) gene from the microalga *Chlorella ellipsoidea*

**DOI:** 10.1186/s12870-017-0995-5

**Published:** 2017-02-21

**Authors:** Xuejie Guo, Chengming Fan, Yuhong Chen, Jingqiao Wang, Weibo Yin, Richard R. C. Wang, Zanmin Hu

**Affiliations:** 1 Institute of Genetics and Developmental Biology, Chinese Academy of Sciences, Beijing, 100101 China; 2 University of Chinese Academy of Sciences, Beijing, 100049 China; 3 Institute of Economical Crops, Yunnan Agricultural Academy, Kunming, 65023 China; 4 United States Department of Agriculture, Agricultural Research Service, Forage and Range Research Laboratory, Utah State University, Logan, UT 84322-6300 USA; 5 Present address: Institute of Genetics and Developmental Biology, Chinese Academy of Sciences, Datun Road, Chaoyang District, Beijing, 100101 China

**Keywords:** *Chlorella ellipsoidea*, Diacylglycerol acyltransferase, Nitrogen starvation, Seed oil content, Seed weight, Triacylglycerol

## Abstract

**Background:**

Oil in the form of triacylglycerols (TAGs) is quantitatively the most important storage form of energy for eukaryotic cells. Diacylglycerol acyltransferase (DGAT) is considered the rate-limiting enzyme for TAG accumulation. *Chlorella*, a unicellular eukaryotic green alga, has attracted much attention as a potential feedstock for renewable energy production. However, the function of DGAT1 in *Chlorella* has not been reported.

**Results:**

A full-length cDNA encoding a putative diacylglycerol acyltransferase 1 (DGAT1, EC 2.3.1.20) was obtained from *Chlorella ellipsoidea*. The 2,142 bp open reading frame of this cDNA, designated *CeDGAT1*, encodes a protein of 713 amino acids showing no more than 40% identity with DGAT1s of higher plants. Transcript analysis showed that the expression level of *CeDGAT1* markedly increased under nitrogen starvation, which led to significant triacylglycerol (TAG) accumulation. CeDGAT1 activity was confirmed in the yeast quadruple mutant strain H1246 by restoring its ability to produce TAG. Upon expression of *CeDGAT1*, the total fatty acid content in wild-type yeast (INVSc1) increased by 142%, significantly higher than that transformed with *DGAT1s* from higher plants, including even the oil crop soybean. The over-expression of Ce*DGAT1* under the NOS promoter in wild-type *Arabidopsis thaliana* and *Brassica napus* var. Westar significantly increased the oil content by 8–37% and 12–18% and the average 1,000-seed weight by 9–15% and 6–29%, respectively, but did not alter the fatty acid composition of the seed oil. The net increase in the 1,000-seed total lipid content was up to 25–50% in both transgenic *Arabidopsis* and *B. napus*.

**Conclusions:**

We identified a gene encoding DGAT1 in *C. ellipsoidea* and confirmed that it plays an important role in TAG accumulation. This is the first functional analysis of DGAT1 in *Chlorella*. This information is important for understanding lipid synthesis and accumulation in *Chlorella* and for genetic engineering to enhance oil production in microalgae and oil plants.

**Electronic supplementary material:**

The online version of this article (doi:10.1186/s12870-017-0995-5) contains supplementary material, which is available to authorized users.

## Background

Triacylglycerols (TAGs) are the main storage lipids in various organisms, such as oilseed plants, oleaginous fungi, yeasts, and microalgae. They are also a major source of highly reduced carbon molecules for food and fuel [1, 2]. TAGs are synthesized in endoplasmic reticulum (ER) and accumulate as oil droplets in lipid bodies, which are generated by budding off from the outer ER membrane [3, 4]. In the Kennedy pathway, TAGs are synthesized by sequentially adding acyl-CoAs to the *sn*-1, *sn*-2 and *sn*-3 positions of a glycerol-3-phosphate molecule [5], which is controlled by four important enzymes, glycerol-3-phosphate acyltransferase (GPAT; EC 2.3.1.15), lyso-phosphatidic acid acyltransferase (LPAT; EC 2.3.1.51), phosphatidate phosphatase (PAP; EC 3.1.3.4) and diacylglycerol acyltransferase (DGAT; EC 3.2.1.20) [6]. DGAT has been proposed to be the rate-limiting enzyme for TAG accumulation [7, 8].

In eukaryotes, three types of DGATs have been reported: the endoplasmic reticulum (ER)- localized DGAT1, DGAT2 and the soluble cytosolic DGAT3. Among them, DGAT1 and DGAT2 are responsible for the bulk of TAG synthesis in most organisms [9]. It has been proposed that these two enzymes have no redundant functions in TAG biosynthesis [10]. DGAT1 plays a dominating role in the determination of oil accumulation and fatty acid composition in seed oils [6], and DGAT2 may influence the content and composition of some plant seed oils containing unusual fatty acids (e.g., epoxy and hydroxyl) [11–13]. The role of the cytosolic DGAT3 has not yet been determined.

DGAT1s are ER membrane-bound proteins and possess six to nine transmembrane domains [14]. The most variable region of DGAT1 is the hydrophilic N terminus, which is quite unique for each DGAT1 and might serve distinct functions in different organisms [15]. Several conserved motifs, including acyl-CoA binding motif, DAG binding motif, the fatty acid-binding protein signature and a putative C-terminal ER retrieval motif, have been identified in DGAT1 [16]. Recently, site-directed mutagenesis was used to demonstrate the importance of some conserved residues in DGAT1s. For instance, mutagenesis at P216 and F439 in *Tropaeolum majus* DGAT1 resulted in a total loss of DGAT1 activity, while the substitution of S197 with alanine in a putative SnRK1 target site resulted in a strong increase in DGAT1 activity in the range of 38% to 80% [6].

The first eukaryotic *DGAT1* gene was cloned from mouse [17], followed by isolation from other organisms [10, 16, 18–26]. Many studies have investigated *DGAT1*s because of their important roles in TAG synthesis and have tried to use them to alter the quality and quantity of storage lipids in higher plants. For instance, the AS11 mutant of *Arabidopsis*, having reduced DGAT activity, showed a 75% reduction in seed lipids, but the expression of *Arabidopsis DGAT1* in the AS11 mutant restored the wild-type levels of TAG and very-long-chain fatty acid content [27]. Moreover, the over-expression of *AtDGAT1* can greatly enhance the TAG content of transformed tobacco [19, 28]. Subsequently, *Tropaeolum majus DGAT1* significantly contributes to seed oil biosynthesis in wild-type *Arabidopsis* and *Brassica napus* by over-expression [6]. The co-expression of an epoxygenase from *Stokesia laevis*, *SlEPX*, and *VgDGAT1* or *VgDGAT2* from *Vernonia galamensis* greatly increased the accumulation of vernolic acid in both petunia leaves and soybean somatic embryos [13]. The over-expression of *DGAT1* from *Jatropha curcas* showed an enhanced total oil content in seeds but did not show any phenotypic differences [25].

Unlike oil crops, microalgae have higher biomass production rates and many are exceedingly rich in oil. Therefore, microalgae have been regarded as potential resources for producing biodiesel, especially neutral lipids (e.g., triacyglycerols; TAGs) [29–31]. Many microalgal strains have the ability to accumulate substantial amounts of lipids in the form of TAGs under stress conditions, such as nitrogen starvation [30]. So far, several *DGAT*s have been cloned and functionally characterized from microalgae. For instance, DGAT1-like [16] and DGAT2B [32] from the diatom *Phaeodactylum tricornutum* have been functionally characterized in a TAG-deficient mutant in the yeast *Saccharomyces cerevisiae*. Furthermore, the over-expression of *PtDGAT2* in *P. tricornutum* resulted in a 35% increase in the neutral lipid content, and the fatty acid composition showed a significant increase in the proportion of polyunsaturated fatty acids [33]. Two DGAT2s (OtDGAT2A and OtDGAT2B) have been identified and characterized from *Ostreococcus tauri*, and OtDGAT2B possesses broad substrate specificity [34]. TpDGAT2 from the marine diatom *Thalassiosira pseudonana* significantly affects the fatty acid profile of TAG [35]. In *Chlamydomonas reinhardtii*, homology searches identified five DGAT2, encoded by *DGTT1*-*DGTT5* [36]. Among them, DGTT1 and DGTT3 are active in TAG synthesis following nitrogen deprivation [37]. The expression of *CrDGTT2* in *Arabidopsis* increased the leaf TAG content, with some molecular species containing very-long-chain fatty acids [38]. A gene encoding DGAT1 was also identified in *C. reinhardtii* after the transcript-based correction of gene models [39]. Other putative *DGAT* genes have been annotated in the genomes of some microalgae, such as *Chlorella variabilis*, *Coccomyxa* sp. C-169, *Volvox carteri* f. *nagariensis*, *Ostreococcus lucimarinus*, *Fragilariopsis cylindrus* and so on [40]. However, to date, there are few reports on the function of DGAT from the unicellular eukaryotic green alga, *Chlorella*, which is a desirable resource for producing biodiesel. Therefore, research on DGAT from *Chlorella* will advance our understanding of the molecular mechanisms underlying lipid metabolism during oil accumulation and will also provide a new means to improve the oil quality and content of microalgae and oil crops.

In the present study, we isolated a *DGAT1* gene (*CeDGAT1*) from *C. ellipsoidea*, a unicellular eukaryotic green alga that can be easily cultured under either autotrophic or heterotrophic conditions, and characterized its function in yeast and higher plants (*Arabidopsis* and *B. napus*). Compared with DGAT1s of higher plants, such as *Glycine max*, *Arabidopsis* and *Brassica oleracea*, CeDGAT1 could more effectively enhance fatty acid accumulation in the wild-type yeast (INVSc1). The over-expression of *CeDGAT1* can significantly enhance the seed oil content and seed weight in *Arabidopsis* and *B. napus*. Furthermore, the expression pattern of the isolated *DGAT1* gene was investigated. This study would be helpful for understanding the function of DGAT from microalgae and for improving oil production in *B. napus*.

## Results

### Identification, sequence and phylogenetic analysis of CeDGAT1 in *C. ellipsoidea*

Based on the expressed sequence tag (EST) data of *C. ellipsoidea*, a full-length cDNA fragment of *C. ellipsoidea DGAT1*, designated as *CeDGAT1*, was cloned and identified. The nucleotide sequence has a full CDS of 2,142 bp, encoding a polypeptide of 713 amino acid residues with a calculated molecular mass of 81.76 kDa. It was registered in GenBank (ID No. KT779429). CeDGAT1 shared no more than 40% identity with DGAT1s of higher plants, such as *G. max* (40%), *Z. mays* (39%), *R. communis* (38%), *Arabidopsis* (38%), *B. napus* (36%), *V. fordii* (35%) and *J. curcas* (34%).

**Fig. 1:**
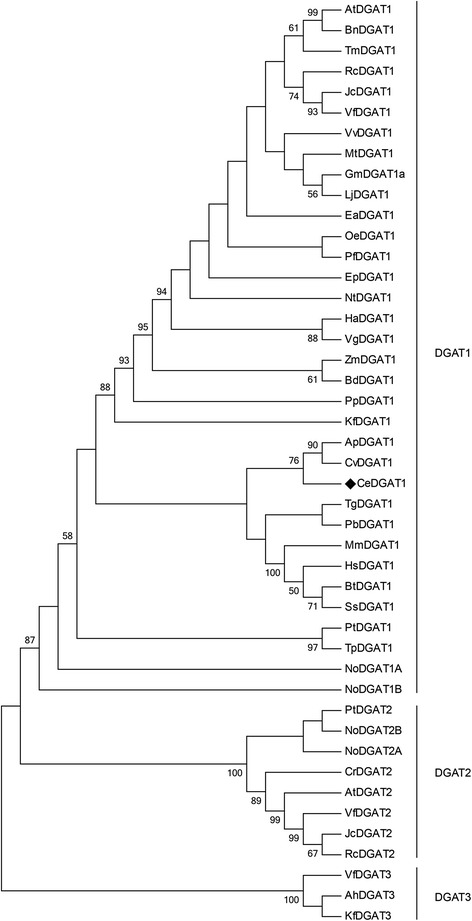
Phylogenetic relationship between CeDGAT1 and DGATs from plants, animals, fungi and microalgae. Sequence alignments were generated with MAFFT, and the phylogenetic tree was constructed by using the the maximum likelihood (ML) method. AtDGAT1 and AtDGAT2 (*Arabidopsis thaliana*, accession no. CAB45373.1 and NP_566952.1, respectively); BnDGAT1 (*Brassica napus*, accession no. AAF64065.1); TmDGAT1 (*Tropaeolum majus*, accession no. AAM03340.2); RcDGAT1 and RcDGAT2 (*Ricinus communis*, accession no. AAR11479.1 and XP_002528531.1, respectively); JcDGAT1 and JcDGAT2 (*Jatropha curcas*, accession no. ABB84383.1 and AFV61670.1); VfDGAT1, VfDGAT2 and VfDGAT3 (*Vernicia fordii*, accession no. ABC94471.1, ABC94473.1 and AGL81309.1, respectively); VvDGAT1 (*Vitis vinifera*, accession no. XP_002279345.1); MtDGAT1 (*Medicago truncatula*, accession no. XP_003595231.1); GmDGAT1a (*Glycine max*, accession no. BAE93460.1); LjDGAT1 (*Lotus japonicas*, accession no. AAW51456.1); EaDGAT1 (*Euonymus alatus*, accession no. AAV31083.1); OeDGAT1 (*Olea europaea*, accession no. AAS01606.1); PfDGAT1 (*Perilla frutescens*, accession no. AAG23696.1); EpDGAT1 (*Echium pitardii*, accession no. ACO55634.1); NtDGAT1 (*Nicotiana tabacum*, accession no. AAF19345.1); HaDGAT1 (*Helianthus annuus*, accession no. ACD67882.1); VgDGAT1 (*Vernonia galamensis*, accession no. ABV21945.1); ZmDGAT1 (*Zea mays*, accession no. ABV91586.1); BdDGAT1 (*Brachypodium distachyon*, accession no. XP_003568769.1); PpDGAT1 (*Physcomitrella patens*, accession no. XP_001770929.1); KfDGAT1 and KfDGAT3 (*Klebsormidium flaccidum*, accession no. GAQ91878.1 and GAQ88368.1, respectively); APDGAT1 (*Auxenochlorella protothecoides*, accession no. XP_011402032.1); CvDGAT1 (*Chlorella vulgaris*, accession no. ALP13863.1); TgDGAT1 (*Toxoplasma gondii*, accession no. AAP94209.1); PbDGAT1 (*Paracoccidioides brasiliensis*, accession no. EEH17170.1); MmDGAT1 (*Mus musculus*, accession no. NP_034176.1); HsDGAT1 (*Homo sapiens*, accession no. NP_036211.2); BtDGAT1 (*Bos Taurus*, accession no. AAL49962.1); SsDGAT1 (*Sus scrofa*, accession no. NP_999216.1); PtDGAT1 and PtDGAT2 (*Phaeodactylum tricornutum*, accession no. ADY76581.1 and AFM37314.1, respectively); TpDGAT1 (*Thalassiosira pseudonana*, accession no. XP_002287215.1); NoDGAT1A, NoDGAT1B, NoDGAT2A and NoDGAT2B (*Nannochloropsis oceanica*) [55]; CrDGAT2 (*Chlamydomonas reinhardtii*, accession no. XP_001693189.1), and AhDGAT3 (*Arachis hypogaea*, accession no. AAX62735.1)

To examine the relationships among different sources of DGAT, a phylogenetic tree was generated from an alignment of the deduced amino acid sequences of CeDGAT1 with 44 DGAT homologues from other species (including membrane-bound DGAT1 and DGAT2 and cytosolic DGAT3). The dendrogram grouped CeDGAT1 with members of the DGAT1 family. DGAT2 and DGAT3 formed a separate cluster different from DGAT1 branches (Fig. 1). In detail, CeDGAT1 clustered together with APDGAT1 (*Auxenochlorella protothecoides*) and CvDGAT1 (*Chlorella vulgaris*) and formed a distinct lineage. Furthermore, CeDGAT1 is more closely related to DGAT1s from animals and fungi than to DGAT1s from plants.

Protein analysis with the TMpred program [41] predicted nine strongly hydrophobic transmembrane regions, which is consistent with the nine transmembrane domains predicted for *B. napus* DGAT1, *T. majus* DGAT1 and *Arabidopsis* DGAT1 [6, 9, 20]. Using the PROSITE database [42], a number of putative functional motifs, including *N*-glycosylation, cAMP- and cGMP-dependent protein kinase phosphorylation, protein kinase C phosphorylation, casein kinase II phosphorylation, tyrosine kinase phosphorylation, *N*-myristoylation and amidation sites were identified in CeDGAT1 (Additional file 1: Table S1). Compared with AtDGAT1, *N*-glycosylation and amidation sites were found only in CeDGAT1, while a leucine zipper pattern was detected only in AtDGAT1. It remains to be determined whether these sites are important for the functional regulation of the enzyme in vivo.

**Fig. 2:**
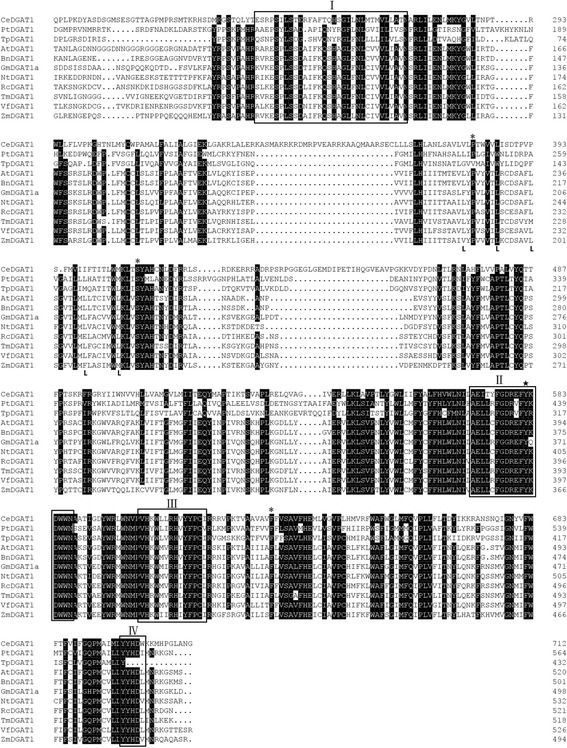
Sequence comparison of CeDGAT1 with DGAT1s from other species. PtDGAT1 (*Phaeodactylum tricornutum*, accession no. ADY76581.1), TpDGAT1 (*Thalassiosira pseudonana*, accession no. XP_002287215.1), AtDGAT1 (*Arabidopsis thaliana*, accession no. CAB45373.1), BnDGAT1 (*Brassica napus*, accession no. AAF64065.1), GmDGAT1a (*Glycine max*, accession no. BAE93460.1), NtDGAT1 (*Nicotiana tabacum*, accession no. AAF19345.1), RcDGAT1 (*Ricinus communis*, accession no. AAR11479.1), TmDGAT1 (*Tropaeolum majus*, accession no. AAM03340.2), VfDGAT1 (*Vernicia fordii*, accession no. ABC94471.1), and ZmDGAT1 (*Zea mays*, accession no. ABV91586.1). Identical amino acid residues are highlighted in black. Conserved motifs or putative signatures are boxed, such as the acyl-CoA binding signature (I); the fatty acid protein signature (II), which contains a tyrosine phosphorylation site (★); the DAG-binding site (III); and the putative endoplasmic reticulum retrieval motif in the C-terminus (IV). The highly conserved proline and serine residues are marked by asterisks. The region containing a conserved leucine repeat (L) is also marked

As previously reported [43, 44], a consensus amino acid sequence for an acyl-CoA binding motif (Fig. 2, I) and a conserved sequence for the DAG-binding motif (Fig. 2, III) of DGAT were also found in CeDGAT1. There was also a fatty acid-binding protein signature spanning residues Ala571 to Asn587 (Fig. 2, II) containing a putative tyrosine phosphorylation site: Tyr582 [6]. The CeDGAT1 protein contained an invariant proline (Pro381), which is thought to participate in presenting the fatty acyl group to the active site for esterification to (diacyl) glycerol and is critical for DGAT1 activity [45]. A highly conserved serine residue (Ser410) was essential for the activity of acyl-CoA: cholesterol acyltransferase, an enzyme closely related to DGAT1 [24, 46]. CeDGAT1 showed a leucine zipper motif with only one conserved leucine (Leu386) in the sequence. A visual examination of CeDGAT1 also revealed the sequence of a putative C-terminal ER retrieval motif (YYHDW, Fig. 2, IV), which is similar to other DGAT1 proteins in plants [11].

### Lipid analysis and expression pattern of *CeDGAT1* in *C. ellipsoidea*

**Fig. 3:**
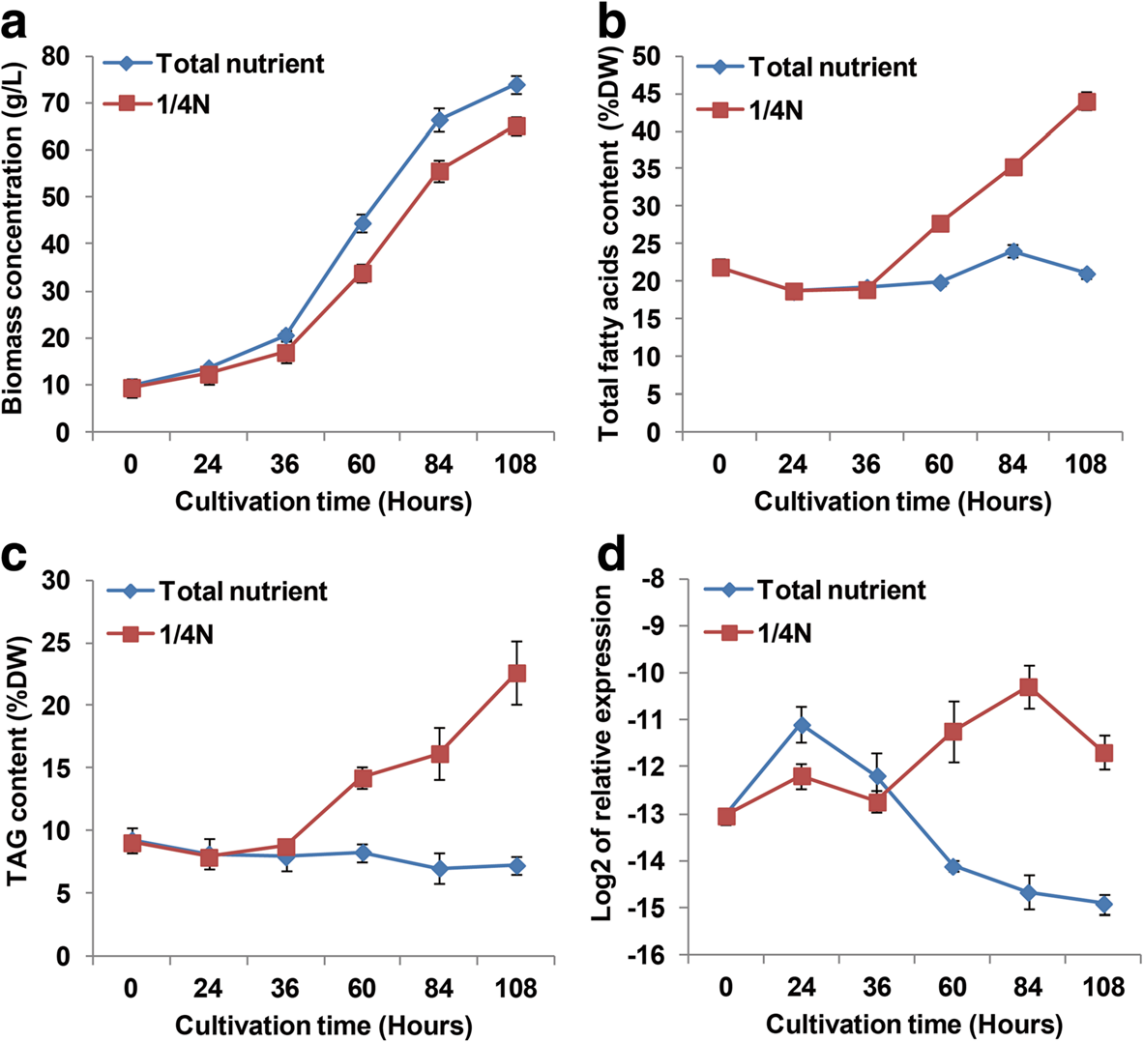
Biomass concentration (**a**), TFA content (**b**), TAG content (**c**) and expression of *CeDGAT1* (**d**) measured from 0 to 108 h in *C. ellipsoidea* cultures grown on nitrogen-replete or nitrogen-depleted (1/4 N) medium. The results are expressed as the mean ± standard deviation (*n* = 3)

To understand the relationship between *CeDGAT1* expression and lipid synthesis, we investigated the expression pattern of *CeDGAT1*, the variation in biomass, and the total fatty acid (TFA) and TAG contents in heterotrophic cultures of *C. ellipsoidea* on nitrogen-replete and nitrogen-depleted (1/4 N) media (Fig. 3). As shown in Fig. 3a, the algal cell biomass increased more slowly in nitrogen-depleted cultures than in nitrogen-replete cultures, but both the TFA content and TAG content increased rapidly from 19% and 9% to 44% and 23%, respectively, from 36 to 108 h (Fig. 3b and c). On the contrary, TFA and TAG contents were not significantly changed in the growth process of nitrogen sufficient condition. So there was a 108% and 212% increase in TFA content and TAG content, respectively at the 108^th^ hour under nitrogen-depleted culture condition than under nitrogen sufficient culture condition.

Quantitative real-time PCR was performed to examine the expression profiles of *CeDGAT1* in *C. ellipsoidea* cells under nitrogen-replete and nitrogen-depleted (1/4 N) conditions (Fig. 3d). 18S *rRNA* was used as an internal reference control. We noted that *CeDGAT1*, which catalyses the last committed step in TAG biosynthesis, was downregulated under nitrogen-replete conditions. Nevertheless, *CeDGAT1* showed transient upregulation, with its transcript level peaking at 84 h following the onset of nitrogen depletion and declining thereafter. The upregulation of *CeDGAT1* was concomitant with the increase in the TFA and TAG contents under nitrogen deprivation, suggesting that *CeDGAT1* was highly induced by nitrogen deprivation and that its increased expression coupled with lipid content change may play an important role in TAG accumulation.

### CeDGAT1 can recover the TAG synthesis of the quadruple mutant yeast strain H1246

To verify the diacylglycerol acyltransferase activity of CeDGAT1, the *CeDGAT1* gene was heterologously expressed in the TAG-deficient *S. cerevisiae* quadruple mutant strain H1246 [47], which lacks the four genes *DGA1*, *LRO1*, *ARE1* and *ARE2* encoding DGAT, PDAT (phosphatidylcholine: diacylglycerol acyltransferase), ASAT1 (acyl-CoA: sterol acyltransferase 1) and ASAT2 (acyl-CoA: sterol acyltransferase 2), respectively. These four genes are essential for the formation of neutral lipids. Lipid bodies can be formed by the expression of at least one of four genes. INVSc1 and H1246 cells harbouring an empty pYES2.0 vector were used as positive and negative controls, respectively.

**Fig. 4:**
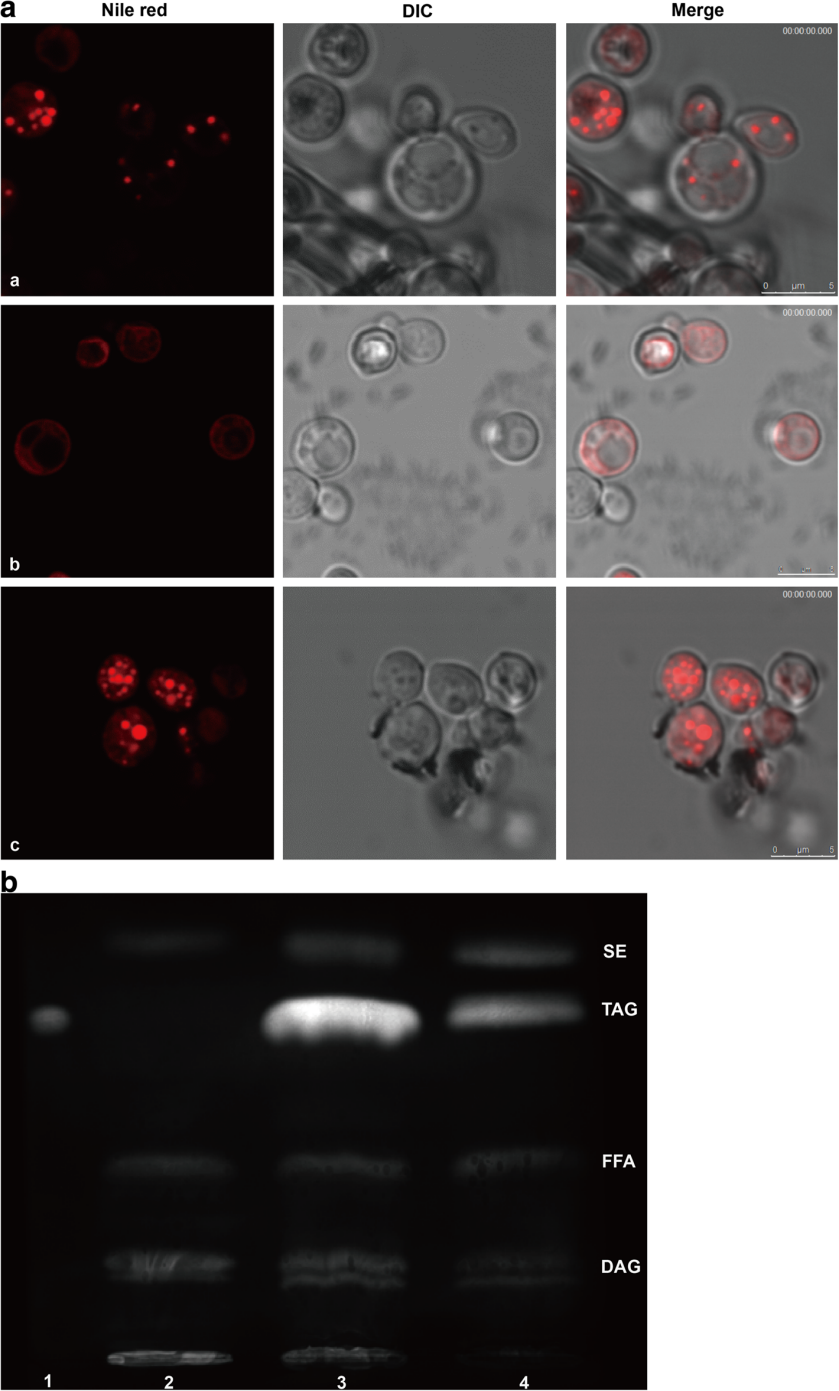
Nile Red staining and TLC analysis of transformed yeast strain H1246. (A) The formation of lipid bodies is restored upon the expression of *CeDGAT1* in the yeast strain H1246. Nile Red staining (left) and interference contrast images (right) of yeast. The wild-type INVSc1 harbouring the empty vector was used as a positive control (a). The mutant strain H1246 harbouring the empty vector was analysed as a negative control (b). The TAG-deficient quadruple mutant strain H1246 expressing *CeDGAT1* (c). Bar = 2.5 μm. (B) TLC analysis of lipid extracts from yeast expressing *CeDGAT1*. (1) TAG marker; (2) The mutant strain H1246 harbouring the empty vector analysed as a negative control; (3) Mutant strain H1246 expressing *CeDGAT1*; and (4) The wild-type strain INVSc1 harbouring the empty plasmid used as a positive control. SE, steryl ester; TAG, Triacylglycerol; FFA, free fatty acid; DAG, diacylglycerol

Nile Red was used to stain lipid bodies in yeast cells. Lipid bodies were present in the wild-type yeast strain INVSc1 or the mutant strain transformed with the *CeDGAT1* gene but were undetectable in the quadruple mutant strain carrying the empty expression vector (pYES2.0) (Fig. 4a). The total lipids were extracted from yeast cells and then subjected to TLC (Thin-Layer Chromatography) analysis. Upon expression of *CeDGAT1*, a prominent band corresponding to TAG appeared on the TLC plate as expected, whereas no TAGs were identified in mutant yeast cells lacking the endogenous yeast DGAT and PDAT activities (Fig. 4b), which is consistent with the results from Nile Red staining (Fig. 4a). These results suggest that CeDGAT1 can successfully restore the ability of the quadruple mutant strain H1246 to form neutral lipids and confirm that *CeDGAT1* encodes a functional protein capable of catalysing the last step of TAG biosynthesis.

### Heterologous expression of *CeDGAT1* can more significantly increase the total fatty acid content in the wild-type yeast than *DGAT1s* from some higher plants

**Fig. 5:**
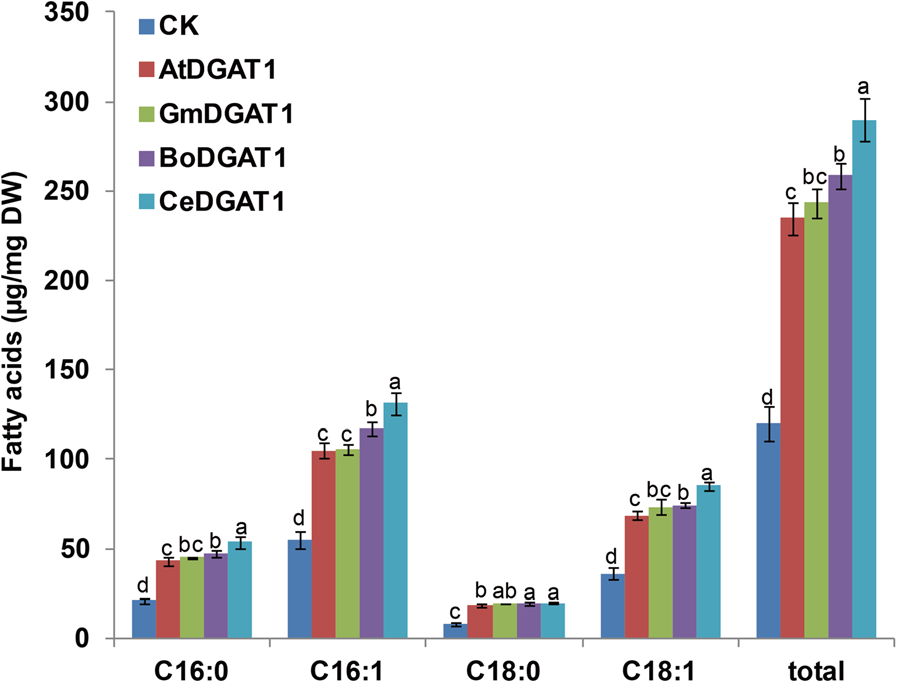
Fatty acids analysis of the wild-type yeast INVSc1 expressing *DGAT1s*. CK, the wild-type yeast INVSc1 transformed with pYES2.0; AtDGAT1, GmDGAT1, BoDGAT1, and CeDGAT1 are transgenic yeast INVSc1 expressing *AtDGAT1*, *GmDGAT1*, *BoDGAT1* and *CeDGAT1*, respectively; C16:0, palmitic acid; C16:1, palmitoleic acid; C18:0, stearic acid; C18:1, oleic acid; and total, the sum of the C16:0, C16:1, C18:0 and C18:1 contents. The bars are the standard deviations (SDs) of three technical repeats. For the same fatty acid component, the numbers with different letters are statistically significant (*P* < 0.05)

We separately transferred *CeDGAT1* and another three *DGAT1* genes from higher plants, including oil crop *G. max*, *A. thaliana* and *B. oleracea*, into the wild-type yeast (INVSc1). RT-PCR results showed that the *DGAT1* genes had nearly identical expression patterns in transgenic yeast (Additional file 2: Figure S1). The fatty acid contents of yeast carrying *DGAT1* genes were measured by GC. As shown in Fig. 5, the total fatty acid contents of yeast significantly increased due to the expression of different *DGAT1*s when compared to the yeast transformed with pYES2.0. In detail, the total fatty acid content in yeast carrying *AtDGAT1*, *GmDGAT1*, *BoDGAT1* and *CeDGAT1* was 234.7 μg/mg, 243.3 μg/mg, 258.5 μg/mg and 290.0 μg/mg, respectively. Among the yeast expressing different *DGAT1* genes, the total fatty acid content in the yeast transformed with *CeDGAT1* increased most remarkably, by 142%. In contrast (compared with the yeast expressing *CeDGAT1*), the total fatty acid contents of the yeasts transformed with the three *DGAT1* genes from higher plants significantly decreased by 19% (*AtDGAT1*), 16% (*GmDGAT1*) and 11% (*BoDGAT1*), respectively. These results suggest that CeDGAT1 may function in improving the oil content of plants, especially oil crops.

### Over-expression of *CeDGAT1* enhances the seed oil content and seed weight in higher plants

**Fig. 6:**
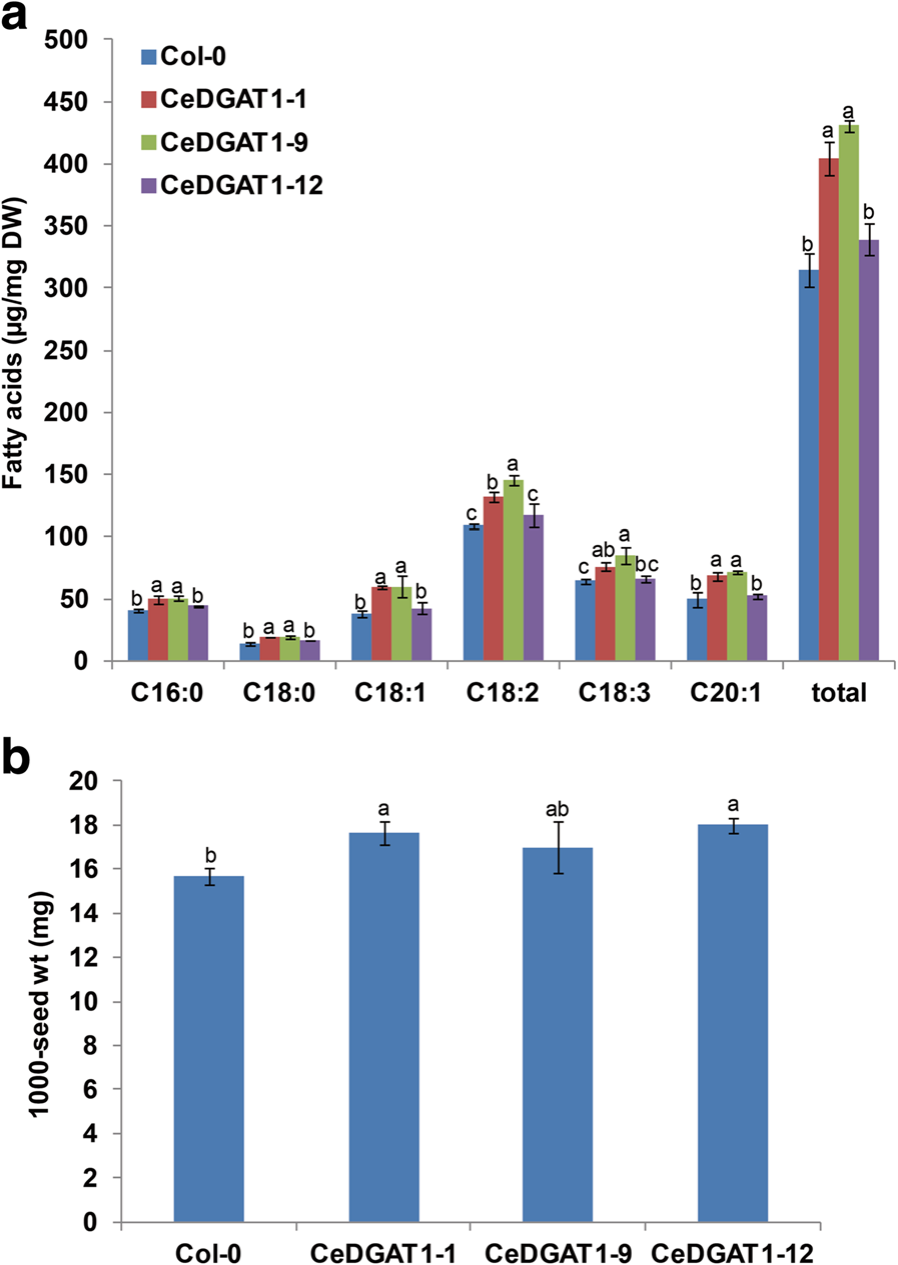
The detection of the fatty acid content and the 1,000-seed weight of *CeDGAT1* transgenic *Arabidopsis*. **a** Average total fatty acid content in dry seeds of different homozygous (T4) transgenic *Arabidopsis* lines. **b** Average 1,000-seed weight (expressed as milligrams of weight/1,000 seeds) of transgenic *Arabidopsis* T4 seeds. Col-0, wild type *Arabidopsis*; CeDGAT1-1, CeDGAT1-9, and CeDGAT1-12, transgenic *Arabidopsis* lines expressing *CeDGAT1*; C16:0, palmitic acid; C18:0, stearic acid; C18:1, oleic acid; C18:2, linoleic acid; C18:3, linolenic acid; C20:1, eicosenoic acid; and total, the sum of the C16:0, C18:0, C18:1, C18:2, C18:3 and C20:1 contents. The bars are the standard deviations (SDs) of three technical repeats. For the same fatty acid component, numbers with different letters are statistically significant (*P* < 0.05)

To explore CeDGAT1 as a tool to manipulate acyl-CoA pools and to engineer TAGs in plants, *CeDGAT1* was over-expressed in *Arabidopsis* and *B. napus* var. Westar under the control of the constitutive NOS promoter. In *Arabidopsis*, three independent homozygous lines were selected for advancement to the T4 generation and used for detailed analysis. RT-PCR results showed that the *CeDGAT1* transcript was expressed in transgenic lines over-expressing *CeDGAT1* (Additional file 3: Figure S2). The transgenic lines did not show any visible morphological difference from untransformed control plants (data not shown). GC analysis revealed that the transformation of wild-type *Arabidopsis* with *CeDGAT1* leads to a higher seed oil content (Fig. 6a). The average total fatty acid content in wild-type *Arabidopsis* seeds was 31.5 mg/100 mg of seed, but increased to 33.9–43.1 mg/100 mg in the transgenic lines. Thus, the transgenic seeds displayed an approximately 8–37% higher oil content than that of wild-type plants. In addition, the 1,000-seed weight of transgenic plants was 9–15% greater than that of the control (Fig. 6b).

**Fig. 7:**
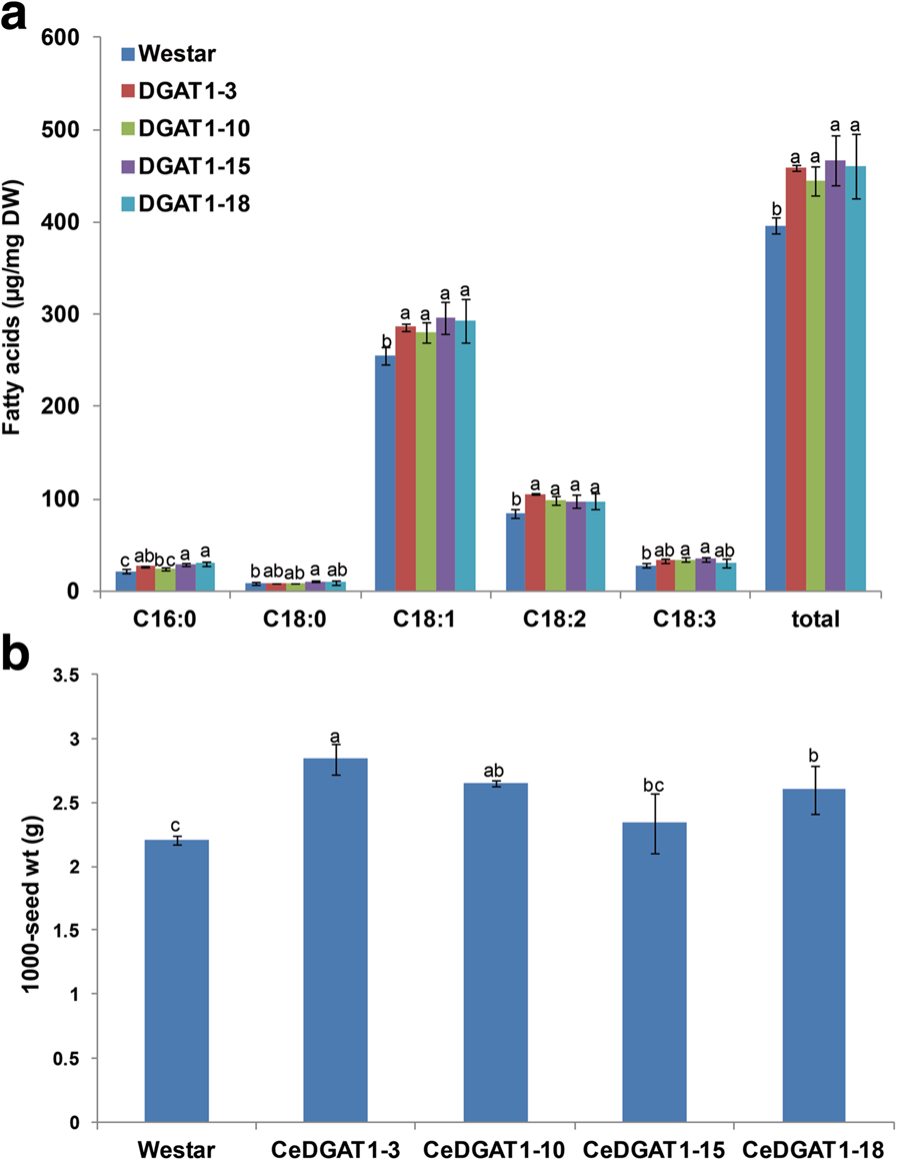
The detection of the fatty acid content and the 1,000-seed weight of *CeDGAT1* transgenic *B. napus*. **a** Average total fatty acid content in dry seeds of different (T3) transgenic *B. napus* lines. **b** Average 1,000-seed weight (expressed as grams of weight/1,000 seeds) of transgenic *B. napus* T3 seeds. Westar, wild type *B. napus* var. Westar; CeDGAT1-3, CeDGAT1-10, CeDGAT1-15, and CeDGAT1-18, transgenic *B. napus* lines expressing *CeDGAT1*; C16:0, palmitic acid; C18:0, stearic acid; C18:1, oleic acid; C18:2, linoleic acid; C18:3, linolenic acid; and total, the sum of the C16:0, C18:0, C18:1, C18:2 and C18:3 contents. The bars are the standard deviations (SDs) of three technical repeats. For the same fatty acid component, numbers with different letters are statistically significant (*P* < 0.05)

In *B. napus*, twelve transgenic plants were identified by PCR (Additional file 4: Figure S3), and four of the twelve independent T3 transgenic *B. napus* lines were chosen for further analysis. The RT-PCR results showed that the *CeDGAT1* transcript was expressed in transgenic lines (Additional file 3: Figure S2). Again, transgenic *B. napus* lines did not show any visible morphological difference from wild-type plants (data not shown). The fatty acid content in the seeds of wild-type *B. napus* and transgenic lines was measured using GC. As shown in Fig. 7a, the average total fatty acid content in wild-type *B. napus* seeds was 39.6 mg/100 mg of seed. In lines expressing *CeDGAT1* under the NOS promoter, the total fatty acid content increased to 44.5–46.8 mg/100 mg of seed, representing an increase of 12–18% over the control. Moreover, the average 1,000-seed weight in the *CeDGAT1* transgenic lines increased by 6–29% compared to that of wild-type plants (Fig. 7b). These results indicated that the *CeDGAT1* gene can stimulate fatty acid biosynthesis and enhance seed weight.

### Subcellular localization of CeDGAT1

**Fig. 8:**
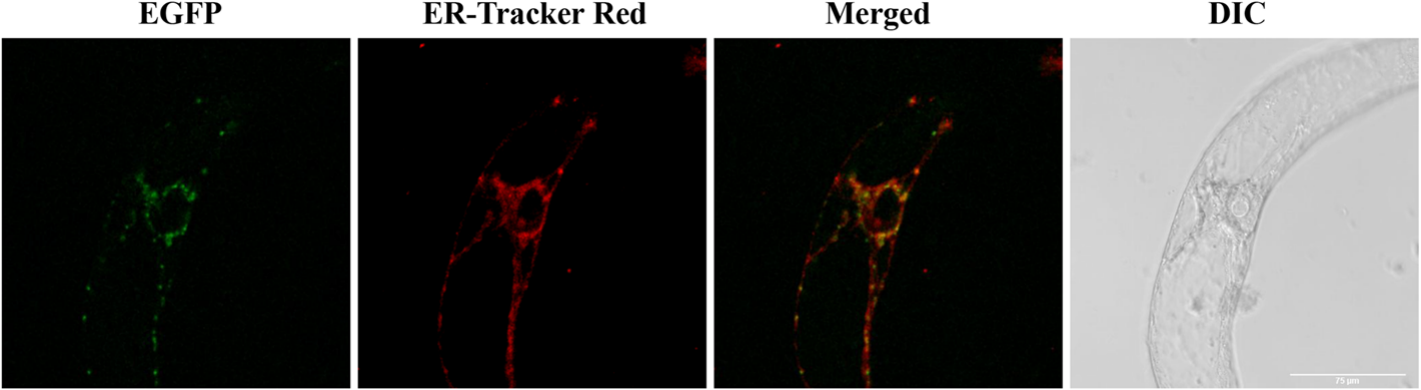
Subcellular localization of CeDGAT1 in BY-2 cells. The endogenous ER in all of the transformed cells was stained with ER-tracker™ Red. Bar = 75 μm

EGFP-tagged CeDGAT1 was expressed in tobacco BY-2 suspension cells and then examined under confocal laser-scanning microscopy. As shown in Fig. 8, the distribution pattern of EGFP-CeDGAT1 was similar to that of endogenous ER stained with ER-tracker™ Red, and the signal was visualized around the nucleus and thread-like ER networks, indicating typical ER localization.

## Discussion

TAGs are quantitatively the most important storage form of energy for eukaryotic cells. The synthesis of TAG from DAG by DGAT is believed to be the major flux control step in oil biosynthesis. Much research has focused on DGAT because it is an enzyme unique to TAG synthesis in plants. However, the function of DGAT1 from *Chlorella* has not been reported.

In this study we cloned and characterized a novel *DGAT1* gene (*CeDGAT1*) from *C. ellipsoidea*. Protein-protein BLAST showed that CeDGAT1 shared no more than 40% identity with DGAT1s of higher plants, which resulted in a difference in the predicted three-dimensional structures (Additional file 5: Figure S4). Functional characterization in yeast showed that CeDGAT1 can increase the TAG content more than can AtDGAT1, GmDGAT1 and BoDGAT1, resulting in a significant increase in the total lipid content of yeast of 142%. Further investigations of the relationships between the CeDGAT1 activity and structure are needed. Its higher activity provides a scientific and economic basis for the use of *C. ellipsoidea* as an oil-producing alga to produce more oil in a short time.

In higher plants, the expression of *DGAT* generally correlates with oil deposition in developing seeds [21]. For soybeans, a stronger expression of *DGAT1* was found in developing seeds than in other tissues [13]. However, DGAT1 transcripts were also detected in other plant tissues, e.g., *AtDGAT* was expressed in a wide range of tissues but most strongly in developing embryos and flower petals [18]. DGAT1 is also highly expressed during pollen development, presumably contributing to TAG accumulation in the pollen grain [48]. These findings suggested that these DGAT enzymes may be related to other physiological processes in addition to seed oil synthesis [15]. For unicellular eukaryotic green algae, all physiological processes take place within a cell; thus, the expression of *DGAT1* can directly reflect the dynamics of TAG accumulation.

The effect of nutrition pattern alteration on algal cell growth, lipid accumulation, and cellular component changes has been analysed in several studies [49–54]. Upon nitrogen starvation, both starches and lipids increased greatly within *C. zofingiensis* [51] and *Nannochloropsis oceanica* cells [54], and N-deficiency plus P-repletion was a promising lipid trigger to motivate lipid accumulation in *C. protothecoides* cells [50]. In *C. reinhardtii*, three genes encoding acyltransferases, *DGAT1*, *DGTT1*, and *PDAT1*, were induced by nitrogen starvation and are likely to play a role in TAG accumulation based on their patterns of expression [39]. At the transcript level in *N. oceanica*, enhanced TAG synthesis under N-depleted conditions primarily involved the upregulation of seven putative *DGAT* genes and the downregulation of six other *DGAT* genes [55]. However, the expression patterns of lipid biosynthesis-related genes, including *DGAT1* in *Chlorella*, have not been extensively studied in these processes. Our results revealed that the upregulation of *CeDGAT1* was closely related to the significant increase in the TFA and TAG contents under nitrogen deprivation, suggesting that *CeDGAT1* plays an important role in TAG accumulation. Our findings contribute to the understanding of the microalgal response to element deprivation and the mechanism of lipid synthesis and accumulation in *Chlorella*, but much remains to be elucidated regarding the precise contribution of N starvation to microalgal metabolism.

Several previous studies have reported that the genetic manipulation of DGAT can lead to increased oil biosynthesis in *Arabidopsis* and *B. napus*. For instance, the seed-specific over-expression of *A. thaliana DGAT1* in wild-type *Arabidopsis* can increase the seed oil content by 11–28% and the seed weight by 2.5–32.3% [43]. Similarly, the seed-specific expression of *TmDGAT1* in wild-type *Arabidopsis* resulted in a 10–33% net increase in the seed oil content and a 15% increase in the 1,000-seed weight in transgenic *Arabidopsis*. Furthermore, the seed-specific expression of *TmDGAT1* in high-erucic acid *B. napus* led to a net increase of 11–15% in the seed oil content of transgenic plants [6]. In addition, the over-expression of *AtDGAT1* and *BnDGAT1* in canola under the control of the napin promoter led to an increase of 2.5–7% in the oil content [56]. The over-expression of *JcDGAT1* in *Arabidopsis* under both CaMV35S promoter and a seed specific promoter resulted in a 30–41% increase in the seed oil content [25]. Our studies showed that the expression of *CeDGAT1* in *Arabidopsis* and *B. napus* under the NOS promoter does indeed increase oil biosynthesis in transgenic seeds by approximately 8–37% and 12–18% over that of the control. In addition, neither the *Arabidopsis* nor the *B. napus CeDGAT1* transformants showed significant changes in fatty acid composition. In some studies, however, there were alterations in the oil composition through *DGAT* expression. The over-expression of *JcDGAT1* in *Arabidopsis* resulted in a significant decrease in oleic acid (C18:1) and an increase in linolenic acid (C18:3) [25], and the transgenic expression of *Sesamum indicum DGAT1* in *Arabidopsis* led to an increase in eicosenoic acid (C20:1) and a reduction in oleic acid (C18:1) in seed oil [26]. More importantly, the expression of *CeDGAT1* in *Arabidopsis* and *B. napus* under the NOS promoter also led to a significant increase in the average 1,000-seed weight in *CeDGAT1* transgenic lines, by 9–15% and 6–29% in *Arabidopsis* and *B. napus*, respectively, and thus there was no decrease in the 1,000-seed weight caused by the oil content increase. Considering the constitutive expression of *CeDGAT1* under the NOS promoter, larger increments in seed oil biosynthesis and seed weight can probably be expected when using a seed specific promoter. Furthermore, there was a difference between *CeDGAT1* transgenic *Arabidopsis* and *B. napus*, with respect to effects on seed oil biosynthesis and seed weight. The seed oil content increased more in transgenic *Arabidopsis* than in *B. napus*, but the average seed weight increase was greater in *B. napus*. Interestingly, the increase in the oil content on a per-1,000-seed basis was similar between transgenic *Arabidopsis* and *B. napus*, at approximately 25–50%. To date, there has been no report that the over-expression of *DGAT1* can significantly increase the seed weight in the oil plant *B. napus*, although this effect has been reported in *Arabidopsis*. In addition, transgenic plants showed no other phenotypic differences. Therefore, CeDGAT1 should have great potential for increasing the net oil production of the oil plant oilseed rape.

## Conclusion

We cloned a novel *DGAT1* gene (*CeDGAT1*) from *C. ellipsoidea*. CeDGAT1 is novel protein, sharing a low identity (≤40%) with DGAT1s from higher plants. The expression of *CeDGAT1* is highly related to rapid lipid accumulation in *C. ellipsoidea* under nitrogen deprivation. In yeast, the expression of *CeDGAT1* can significantly increase the lipid content and shows greater ability for improving the lipid synthesis than *DGAT1s* from some higher plants, including that from soybean. Moreover, the expression of *CeDGAT1* in *Arabidopsis* and oilseed rape can lead to a net increase in the 1,000-seed lipid content of transgenic plants of 25–50%. These findings should be helpful for understanding the function of DGAT from microalgae and the mechanism of lipid synthesis and accumulation and may also provide technology for enhancing lipid production in microalgae and oil plants.

## Methods

### Strains and growth conditions

*Chlorella ellipsoidea* was initially cultured mixotrophically in 1 L flasks containing 500 mL of sterilized Endo medium [57] and incubated at 25 °C under illumination (100 μmol photons/m^2^/s) for one week with shaking at 160 r/min. These pre-cultured cells were transferred to Erlenmeyer flasks (3 L), each containing 1 L of fresh medium to a final volume of 1.5 L, and incubated for another 4 days. These pre-cultured cells, after centrifugation and washing with sterilized water, were sampled as the starting point (0 h). Then, the collected cells were resuspended at a density of approximately 9.5 g/L in a 20 L BioFlo 415 fermentor (New Brunswick Scientific, USA) containing 14 L of modified Endo medium, in which urea was used to replace KNO_3_ in the original Endo medium. The media containing urea at 0.4 g/L and 1.6 g/L were named N-depleted (1/4 N) and N-repletion media, respectively. The culture conditions were maintained at 25 °C, and a thermocirculator was used to maintain a constant temperature in the fermentor by circulating water through the jacket. The fermentor was aerated with filtered ambient air at a flow rate of 0.5 vvm, and the pH was maintained at 6.8 using 1 M KOH. The cultures were sampled 24, 36, 60, 84 and 108 h after they were initiated using N-depleted (1/4 N) or N-repletion medium. The cells were harvested by centrifugation (5,000 g at 4 °C for 5 min). Aliquots for RNA analysis and gene cloning were frozen in liquid nitrogen and stored at −80 °C if not immediately used, while those for lipid analysis were washed with water and freeze-dried.

The wild-type yeast strain INVSc1 (Invitrogen, UK), the H1246 mutant strain (*Matαyor245c::KanMX4 lro1::TRP1 are1::HIS3 are2::LEU2 ADE2 ura3*) [47], *Arabidopsis thaliana* (ecotype Columbia) and *Brassica napus* (Westar) were used to determine the function of CeDGAT1 by heterologous expression.

### Cloning of a cDNA encoding DGAT1 from *C. ellipsoidea*

Total RNA was isolated from algae cells of the exponential growth phase of *C. ellipsoidea* using the EasySpin RNA Extraction Kit (Aidlab Biotech, Beijing, China), and cDNA was prepared from 5 μg of total RNA-template with the ReverTra Ace qPCR RT Kit (Toyobo, Osaka, Japan). The coding sequence of CeDGAT1 was amplified using the gene-specific primers P1 and P2 (Additional file 6: Table S2) based on the expressed sequence tag (EST) data of *C. ellipsoide*a. The 25 μL final reaction volume used for PCR contained 2.5 μL of 10× PCR buffer with MgCl_2_, 1 μL of each primer (10 μM), 2.0 μL of 2.5 mM dNTPs, 1 μL of cDNA sample, 0.5 μL of EasyPfu DNA polymerase (TransGen Biotech, Beijing, China), and 17 μL of double-distilled water. The reaction conditions for PCR were as follows: denatured at 95 °C for 10 min, followed by 30 cycles of 94 °C for 30 s, 55 °C for 30 s, and 72 °C for 2 min; and a final extension step of 72 °C for 10 min. The amplified cDNA was cloned into the *pEASY*-Blunt vector (TransGen Biotech, Beijing, China), and the corresponding clones were verified by PCR and DNA sequencing.

### Yeast expression vector construction and transformation

The full-length *CeDGAT1* ORF was amplified using primers P3 and P4 (Additional file 6: Table S2) and subcloned between the *Hin*d III and *Eco*R I sites of the pYES2.0 yeast expression vector. The *Saccharomyces cerevisiae* strains (the wild-type strain INVSc1 and the mutant strain H1246) were transformed using the LiAc method [58]. We also separately transferred another three *DGAT1* genes from higher plants, including the oil crop *Glycine max* (accession no. AY496439.1), *Arabidopsis thaliana* (accession no. NM_127503.2) and *Brassica oleracea* (unpublished data from our laboratory) into the wild-type yeast (INVSc1). The primers that were used for *DGAT1* gene cloning are shown in Additional file 6: Table S2. Transformants were selected on synthetic complete medium lacking uracil (SC-ura). For heterologous expression studies, the yeast strains were transferred into liquid SC-ura medium containing 2% (w/v) glucose at 30 °C overnight and then induced by adding 2% (w/v) galactose and 1% (w/v) tergitol NP-40 (Sigma, Taufkirchen, Germany) for an additional 72 h at 20 °C. The expression of *DGAT1*s in transgenic yeast was verified at the transcript level by RT-PCR (for the RT-PCR primers see Additional file 6: Table S2).

### Plant vector construction and transformation

The complete *CeDGAT1* was cloned into the plant expression vector pCAMBIA2301 under the control of the nopaline synthase (NOS) promoter and nos terminator, yielding pCAMBIA2301-*CeDGAT1* (Additional file 7: Figure S5). The final binary vector was verified and then transferred into *Agrobacterium tumefaciens* strain GV3101 by the freeze-thaw method [59]. *Arabidopsis* plants were transformed by vacuum infiltration [60]. *Brassica napus* var. Westar was transformed using hypocotyl explants and the modified method of DeBlock et al. [61]. T1 generation seeds were selected on kanamycin (50 mg/L), and then the selected transformed plants were transferred to soil. T3 transgenic *B. napus* lines and homozygous T4 transgenic *Arabidopsis* lines were used for seed and oil analyses. Genomic DNA was isolated from *B. napus* var. Westar leaf material. The stable integration of the NOS: CeDGAT1: nos cassette into the genome of transgenic *B. napus* was checked by PCR amplification using the specific primers P21 and P22 (Additional file 6: Table S2). GUS histochemical staining of the leaves from the transgenic lines was also conducted as described by Jefferson et al. [62]. In the meantime, the expression of *CeDGAT1* in *Arabidopsi*s and *B. napus* was detected by RT-PCR using the *CeDGAT1-*specific primers P11 and P12 (Additional file 6: Table S2). The *Arabidopsis* housekeeping gene *actin* (primers P23 and P24) and the *B. napus* housekeeping gene *GAPDH* (primers P25 and P26) were used as internal controls.

### Nile Red staining and microscopy

The Nile Red staining was used to visualize the intracellular lipid bodies as an indicators of TAG formation [63]. For yeast cell staining, a 500 μL suspension of yeast cells in the culture medium was stained with 5 μL of Nile Red (1 mg/mL in acetone stock), incubated in the dark for 5 min, and immediately used for microscopic analysis.

### Lipid analysis by TLC and GC-MS

For the analysis of lipids from yeast and *C. ellipsoidea*, the cells were harvested by centrifugation, and the resulting cell pellets were ground to a fine powder under liquid nitrogen and subsequently treated with isopropanol at 80 °C for 10–15 min to stop the lipolytic activity. Isopropanol was evaporated under nitrogen gas before lipid extraction. The total lipids were extracted according to a modified version of the Bligh and Dyer method [64], and TAG was separated from the total lipids by thin-layer chromatography (TLC) on Silica Gel 60 plates (Merck, Darmstadt, Germany). The solvents that were used were hexane/diethyl ether/glacial acetic acid (70:30:1, v/v). The lipids were visualized by spraying Primuline (Sigma, 10 mg/100 mL acetone: water (60:40 v/v)) and exposing the plate to UV. Triolein (Sigma) was used as the standard. TAGs were recovered from the TLC plates and then trans-esterified with 5% H_2_SO_4_ in methanol at 85 °C for 1 h. The fatty acid methyl esters (FAMEs) were extracted with hexane and analysed by GC-MS following the methods described in the following section.

### Fatty acid analysis

Cellular fatty acids were extracted by incubating 10 mg of dried seeds of control and transformed plants or 50 mg of yeast powder and freeze-dried algae powder in 3 mL of 7.5% (w/v) KOH in methanol for saponification at 70 °C for 4 h. After the pH was adjusted to 2.0 with HCl, the fatty acid were subjected to methylesterification with 2 mL of 14% (w/v) boron trifluoride in methanol at 70 °C for 1.5 h. A phase separation was produced by adding 1 mL of 0.9% (w/v) NaCl and 4 mL of hexane. The upper phase was dried under a nitrogen gas flow and resuspended in 0.3 mL of acetic ether prior to GC analysis. An analysis of fatty acid methyl esters (FAME) was performed by GC-MS (gas chromatography–mass spectrometry, TurboMass, PerkinElmer, USA) equipped with a capillary column (BPX-70, 30 m × 0.25 mm × 0.25 μm). Hydrogen was used as the carrier gas at a flow rate of 1.0 mL/min. The injector and detector temperatures were held at 250 °C. The column oven was temperature-programmed from 100 to 190 °C at 15 °C/min, where the temperature was held for 1 min increased to 220 °C at 10 °C/min, and then held for 4 min. The total FA content was quantified using heptadecanoic acid (C17:0) (Sigma) as an internal standard added to samples prior to extraction.

### Dry weight determination

For dry weight determination, the algal cells were collected by filtering the culture through pre-weighed Whatman GF/C filter paper (1.2 μm pore size). Then, the filter paper was dried at 80 °C in an oven until the weight was constant.

The *C. ellipsoidea* biomass concentration (w/v) was equivalent to a specific value of the cell dry weight (DW) that was determined by OD_540_ according to the following empirical formula:

$$ \mathrm{D}\mathrm{W}\ \left(\mathrm{g}/\mathrm{L}\right) = \left(\mathrm{O}{\mathrm{D}}_{540} + 0.0097\right)/0.4165 $$

### Quantitative real-time PCR detection of *CeDGAT1* expression in *C. ellipsoidea*

The total RNA was isolated from the cells of *C. ellipsoidea* at six growing points (0 h, 24 h, 36 h, 60 h, 84 h, and 108 h) during the time course of nitrogen depletion or repletion. All of the real-time reactions were performed on a LightCycler® 480 Real-Time PCR System (Roche Applied Science, Mannheim, Germany) using the LightCycler® 480 SYBR Green I Master Mix Kit (Roche Applied Science) according to the manufacturer’s instructions: 95 °C for 30 s and then 40 cycles of 95 °C for 10 s, followed by 55 °C for 10 s, and 72 °C for 20 s. All of the qRT-PCR experiments were performed in triplicate. The primers that were used for the qRT-PCR of *CeDGAT1* are P27 and P28 (Additional file 6: Table S2). To normalize the transcript levels in each sample, 18S *rRNA* was used as the internal standard (primers P29 and P30). The relative expression was computed following the formula (Cta-Ctb), where Cta and Ctb are the average Ct values of the reference and target genes, respectively.

### Alignment and molecular phylogenetic analysis

Multiple alignments were performed using MAFFT v6.847b [65] with the L-INS-i algorithm. A phylogenetic tree was reconstructed with FastTree using the approximate maximum-likelihood method [66]. For testing the robustness of the tree, 1000 bootstrap replicates were carried out. The transmembrane regions in the CeDGAT1 protein were predicted with the TMpred program (http://www.ch.embnet.org/software/TMPRED_form.html). Three-dimensional structures of the DGAT1 proteins (CeDGAT1, AtDGAT1, GmDGAT1, and BoDGAT1) were predicted by I-TASSER (http://zhanglab.ccmb.med.umich.edu/I-TASSER/).

### Subcellular localization of CeDGAT1 in tobacco BY-2 Cells

To determine its subcellular location, *CeDGAT1* without the stop codon was amplified from cDNA, cloned into the GATEWAY donor vector pGWC according to the method described by Chen et al. [67] and sequenced. The *CeDGAT1* was then introduced in the destination vector pGWB5 with infusion with EGFP under the CaMV 35S promoter by LR reaction following the manufacturer’s instructions (Invitrogen). The final construct CeDGAT1::EGFP was transferred into *Agrobacterium tumefaciens* strain GV3101. The *Agrobacterium*-mediated transformation of tobacco BY2 cells was performed according to An [68] and Genschik et al. [69]. Briefly, BY2 cells were incubated for 3 days at 27 °C in the dark (without shaking) with *Agrobacterium* GV3101 containing CeDGAT1::EGFP. Subsequently, cells were plated on medium containing two antibiotics: timentin (500 mg/L) to kill off *Agrobacterium* and kanamycin (100 mg/L) to select for transformed cells. Transformed cells appeared after one month as a callus on plates and were then transferred to fresh plates once a month. A suspension culture was obtained by the addition of small transformed callus clumps to liquid culture medium containing kanamycin. The transformed BY-2 suspension cells were stained with ER-tracker™ Red following the manufacturer’s instructions (Invitrogen) and then observed and photographed using a Leica TCS SP5 confocal laser scanning microscope (Leica Microsystems, Germany).

### Statistical analysis

All of the experimental data were statistically compared using a one-way analysis of variance (ANOVA) with the software Statistical Product and Service Solutions (SPSS) v19.0, followed by a post-hoc test to determine the significant difference among the treatment means.

## Additional files

Additional file 1: Table S1. Putative functional motifs in CeDGAT1 and AtDGAT1. (DOCX 17 kb)
Additional file 2: Figure S1. RT-PCR detection of *DGAT1* genes in transgenic yeast (INVSc1). The yeast *actin* was used as an internal control. 1, The yeast transformed with pYES2.0; 2–5, the yeast expressing *AtDGAT1*, *GmDGAT1*, *BnDGAT1* and *CeDGAT1*, respectively. (DOCX 55 kb)
Additional file 3: Figure S2. A schematic map of the pCAMBIA2301-NOS-CeDGAT1-nos plasmid. The pCAMBIA2301-NOS-CeDGAT1-nos vector contained an expression box of the *CeDGAT1* gene from *C. ellipsoidea* under the control of the NOS promoter and nos terminator; an expression box of the *GUS* gene controlled by the CaMV35S promoter and nos terminator; and an expression box of the kanamycin resistance gene, which conferred resistance to kanamycin. (DOCX 243 kb)
Additional file 4: Figure S3. RT-PCR detection of Ce*DGAT1* in transgenic *Arabidopsis* (A) and *B. napus* (B) lines. *Arabidopsis actin* and *B. napus GAPDH* were used as internal controls. Col-0, wild-type *Arabidopsis*; 1, 9, and 12, transgenic *Arabidopsis* lines expressing NOS:CeDGAT1; Westar, wild-type *B. napus* var. Westar; 3, 10, 15, and 18, transgenic *B. napus* lines expressing NOS:CeDGAT1. (DOCX 93 kb)
Additional file 5: Figure S4. PCR detection of the *CeDGAT1* gene in transgenic *B. napus* lines. M, DNA molecular weight marker; 1, blank control; 2, wild-type *B. napus* var. Westar; 3–14, lines expressing NOS:CeDGAT1; 15, pCAMBIA2301-NOS-CeDGAT1-nos plasmid (positive control). (DOCX 112 kb)
Additional file 6: Table S2. List of primers. Sequences in lower-case letters indicate enzyme restriction sites. (DOCX 14 kb)
Additional file 7: Figure S5. Comparison of the predicted structures of CeDGAT1, AtDGAT1, GmDGAT1 and BoDGAT1. (DOCX 416 kb)

## References

[1] Durrett TP, Benning C, Ohlrogge J (2008). Plant triacylglycerols as feedstocks for the production of biofuels. Plant J.

[2] Dyer JM, Stymne S, Green AG, Carlsson AS (2008). High-value oils from plants. Plant J.

[3] Huang AHC (1996). Oleosins and oil bodies in seeds and other organs. Plant Physiol.

[4] Chapman KD, Dyer JM, Mullen RT (2012). Biogenesis and functions of lipid droplets in plants: thematic review series: lipid droplet synthesis and metabolism: from yeast to man. J Lipid Res.

[5] Nicole K, Simoni RD, Hill RL (2005). Otto Fritz Meyerhof and the elucidation of the glycolytic pathway. J Biol Chem.

[6] Xu J, Francis T, Mietkiewska E, Giblin EM, Barton DL, Zhang Y (2008). Cloning and characterization of an acyl-CoA-dependent diacylglycerol acyltransferase 1 (DGAT1) gene from *Tropaeolum majus*, and a study of the functional motifs of the DGAT protein using site-directed mutagenesis to modify enzyme activity and oil content. Plant Biotechnol J.

[7] Ichihara KI, Takahashi T, Fujii S (1988). Diacylglycerol acyltransferase in maturing safflower seeds: its influences on the fatty acid composition of triacylglycerol and on the rate of triacylglycerol synthesis. Biochim Biophys Acta.

[8] Settlage SB, Kwanyuen P, Wilson RF (1998). Relation between diacylglycerol acyltransferase activity and oil concentration in soybean. J Am Oil Chem Soc.

[9] Turchetto-Zolet AC, Maraschin FS, de Morais GL, Cagliari A, Andrade CM, Margis-Pinheiro M (2011). Evolutionary view of acyl-CoA diacylglycerol acyltransferase (DGAT), a key enzyme in neutral lipid biosynthesis. BMC Evol Biol.

[10] Shockey JM, Gidda SK, Chapital DC, Kuan JC, Dhanoa PK, Bland JM (2006). Tung tree DGAT1 and DGAT2 have nonredundant functions in triacylglycerol biosynthesis and are localized to different subdomains of the endoplasmic reticulum. Plant Cell.

[11] Kroon JT, Wei W, Simon WJ, Slabas AR (2006). Identification and functional expression of a type 2 acyl-CoA:diacylglycerol acyltransferase (DGAT2) in developing castor bean seeds which has high homology to the major triglyceride biosynthetic enzyme of fungi and animals. Phytochemistry.

[12] Cahoon EB, Shockey JM, Dietrich CR, Gidda SK, Mullen RT, Dyer JM (2007). Engineering oilseeds for sustainable production of industrial and nutritional feedstocks: solving bottlenecks in fatty acid flux. Curr Opin Plant Biol.

[13] Li R, Yu K, Hatanaka T, Hildebrand DF (2010). Vernonia DGATs increase accumulation of epoxy fatty acids in oil. Plant Biotechnol J.

[14] Yen CLE, Stone SJ, Koliwad S, Harris C, Farese RV (2008). DGAT enzymes and triacylglycerol biosynthesis. J Lipid Res.

[15] Liu Q, Siloto RM, Lehner R, Stone SJ, Weselake RJ (2012). Acyl-CoA:diacylglycerol acyltransferase: molecular biology, biochemistry and biotechnology. Prog Lipid Res.

[16] Guiheneuf F, Leu S, Zarka A, Khozin-Goldberg I, Khalilov I, Boussiba S (2011). Cloning and molecular characterization of a novel acyl-CoA:diacylglycerol acyltransferase 1-like gene (*PtDGAT1*) from the diatom *Phaeodactylum tricornutum*. FEBS J.

[17] Cases S, Novak S, Zheng YW, Myers HM, Lear SR, Sande E (1998). ACAT-2, a second mammalian acyl-CoA : cholesterol acyltransferase - its cloning, expression, and characterization. J Biol Chem.

[18] Hobbs DH, Lu CF, Hills MJ (1999). Cloning of a cDNA encoding diacylglycerol acyltransferase from *Arabidopsis thaliana* and its functional expression. FEBS Lett.

[19] Bouvier-Nave P, Benveniste P, Oelkers P, Sturley SL, Schaller H (2000). Expression in yeast and tobacco of plant cDNAs encoding acyl CoA : diacylglycerol acyltransferase. Eur J Biochem.

[20] Nykiforuk CL, Furukawa-Stoffer TL, Huff PW, Sarna M, Laroche A, Moloney MM (2002). Characterization of cDNAs encoding diacylglycerol acyltransferase from cultures of *Brassica napus* and sucrose-mediated induction of enzyme biosynthesis. Biochim Biophys Acta.

[21] He XH, Turner C, Chen GQ, Lin JT, McKeon TA (2004). Cloning and characterization of a cDNA encoding diacylglycerol acyltransferase from castor bean. Lipids.

[22] Milcamps A, Tumaney AW, Paddock T, Pan DA, Ohlrogge J, Pollard M (2005). Isolation of a gene encoding a 1,2-diacylglycerol-*sn*-acetyl-CoA acetyltransferase from developing seeds of *Euonymus alatus*. J Biol Chem.

[23] Wang HW, Zhang JS, Gai JY, Chen SY (2006). Cloning and comparative analysis of the gene encoding diacylglycerol acyltransferase from wild type and cultivated soybean. Theor Appl Genet.

[24] Yu K, Li R, Hatanaka T, Hildebrand D (2008). Cloning and functional analysis of two type 1 diacylglycerol acyltransferases from *Vernonia galamensis*. Phytochemistry.

[25] Misra A, Khan K, Niranjan A, Nath P, Sane VA (2013). Over-expression of *JcDGAT1* from *Jatropha curcas* increases seed oil levels and alters oil quality in transgenic *Arabidopsis thaliana*. Phytochemistry.

[26] Wang Z, Huang W, Chang J, Sebastian A, Li Y, Li H (2014). Overexpression of *SiDGAT1*, a gene encoding acyl-CoA:diacylglycerol acyltransferase from *Sesamum indicum* L. increases oil content in transgenic *Arabidopsis* and soybean. Plant Cell Tissue Organ Cult..

[27] Katavic V, Reed DW, Taylor DC, Giblin EM, Barton DL, Zou JT (1995). Alteration of seed fatty-acid composition by an ethyl methanesulfonate-induced mutation in *Arabidopsis thaliana* affecting diacylglycerol acyltransferase activity. Plant Physiol.

[28] Andrianov V, Borisjuk N, Pogrebnyak N, Brinker A, Dixon J, Spitsin S (2010). Tobacco as a production platform for biofuel: overexpression of *Arabidopsis DGAT* and *LEC2* genes increases accumulation and shifts the composition of lipids in green biomass. Plant Biotechnol J.

[29] Chisti Y (2007). Biodiesel from microalgae. Biotechnol Adv.

[30] Hu Q, Sommerfeld M, Jarvis E, Ghirardi M, Posewitz M, Seibert M (2008). Microalgal triacylglycerols as feedstocks for biofuel production: perspectives and advances. Plant J.

[31] Lam MK, Lee KT (2012). Microalgae biofuels: a critical review of issues, problems and the way forward. Biotechnol Adv.

[32] Gong Y, Zhang J, Guo X, Wan X, Liang Z, Hu CJ (2013). Identification and characterization of PtDGAT2B, an acyltransferase of the DGAT2 acyl-coenzyme A: diacylglycerol acyltransferase family in the diatom *Phaeodactylum tricornutum*. FEBS Lett.

[33] Niu YF, Zhang MH, Li DW, Yang WD, Liu JS, Bai WB (2013). Improvement of neutral lipid and polyunsaturated fatty acid biosynthesis by overexpressing a type 2 diacylglycerol acyltransferase in marine diatom *Phaeodactylum tricornutum*. Mar Drugs.

[34] Wagner M, Hoppe K, Czabany T, Heilmann M, Daum G, Feussner I (2010). Identification and characterization of an acyl-CoA:diacylglycerol acyltransferase 2 (*DGAT2*) gene from the microalga *O. tauri*. Plant Physiol Biochem.

[35] Xu J, Kazachkov M, Jia Y, Zheng Z, Zou J (2013). Expression of a type 2 diacylglycerol acyltransferase from *Thalassiosira pseudonana* in yeast leads to incorporation of docosahexaenoic acid β-oxidation intermediates into triacylglycerol. FEBS J.

[36] Miller R, Wu G, Deshpande RR, Vieler A, Gartner K, Li X (2010). Changes in transcript abundance in *Chlamydomonas reinhardtii* following nitrogen deprivation predict diversion of metabolism. Plant Physiol.

[37] La Russa M, Bogen C, Uhmeyer A, Doebbe A, Filippone E, Kruse O (2012). Functional analysis of three type-2 DGAT homologue genes for triacylglycerol production in the green microalga *Chlamydomonas reinhardtii*. J Biotechnol.

[38] Sanjaya MR, Durrett TP, Kosma DK, Lydic TA, Muthan B (2013). Altered lipid composition and enhanced nutritional value of Arabidopsis leaves following introduction of an algal diacylglycerol acyltransferase 2. Plant Cell.

[39] Boyle NR, Page MD, Liu B, Blaby IK, Casero D, Kropat J (2012). Three acyltransferases and nitrogen-responsive regulator are implicated in nitrogen starvation-induced triacylglycerol accumulation in *Chlamydomonas*. J Biol Chem.

[40] Chen JE, Smith AG (2012). A look at diacylglycerol acyltransferases (DGATs) in algae. J Biotechnol.

[41] Hofman K (1993). Tmbase-a database of membrane spanning protein segments. Biol. Chem. Hoppe Seyler.

[42] Hulo N, Bairoch A, Bulliard V, Cerutti L, De Castro E, Langendijk-Genevaux PS (2006). The PROSITE database. Nucleic Acids Res.

[43] Jako C, Kumar A, Wei YD, Zou JT, Barton DL, Giblin EM (2001). Seed-specific over-expression of an *Arabidopsis* cDNA encoding a diacylglycerol acyltransferase enhances seed oil content and seed weight. Plant Physiol.

[44] Manas-Fernandez A, Vilches-Ferron M, Garrido-Cardenas JA, Belarbi EH, Alonso DL, Garcia-Maroto F (2009). Cloning and molecular characterization of the acyl-CoA: diacylglycerol acyltransferase 1 (*DGAT1*) gene from *Echium*. Lipids.

[45] Lewin TM, Wang P, Coleman RA (1999). Analysis of amino acid motifs diagnostic for the sn-glycerol-3-phosphate acyltransferase reaction. Biochemistry.

[46] Joyce CW, Shelness GS, Davis MA, Lee RG, Skinner K, Anderson RA (2000). ACAT1 and ACAT2 membrane topology segregates a serine residue essential for activity to opposite sides of the endoplasmic reticulum membrane. Mol Biol Cell.

[47] Sandager L, Gustavsson MH, Stahl U, Dahlqvist A, Wiberg E, Banas A (2002). Storage lipid synthesis is non-essential in yeast. J Biol Chem.

[48] Zhang M, Fan JL, Taylor DC, Ohlrogge JB (2009). DGAT1 and PDAT1 acyltransferases have overlapping functions in *Arabidopsis* triacylglycerol biosynthesis and are essential for normal pollen and seed development. Plant Cell.

[49] Illman AM, Scragg AH, Shales SW (2000). Increase in *Chlorella* strains calorific values when grown in low nitrogen medium. Enzym. Microb. Technol..

[50] Li Y, Han F, Xu H, Mu J, Chen D, Feng B (2014). Potential lipid accumulation and growth characteristic of the green alga *Chlorella* with combination cultivation mode of nitrogen (N) and phosphorus (P). Bioresour. Technol..

[51] Zhu S, Huang W, Xu J, Wang Z, Xu J, Yuan Z (2014). Metabolic changes of starch and lipid triggered by nitrogen starvation in the microalga *Chlorella zofingiensis*. Bioresour. Technol..

[52] Cakmak T, Angun P, Ozkan AD, Cakmak Z, Olmez TT, Tekinay T (2012). Nitrogen and sulfur deprivation differentiate lipid accumulation targets of *Chlamydomonas reinhardtii*. Bioengineered.

[53] Ho SH, Chen CY, Chang JS (2012). Effect of light intensity and nitrogen starvation on CO_2_ fixation and lipid/carbohydrate production of an indigenous microalga *Scenedesmus obliquus* CNW-N. Bioresour. Technol..

[54] Jia J, Han D, Gerken HG, Li Y, Sommerfeld M, Hu Q (2015). Molecular mechanisms for photosynthetic carbon partitioning into storage neutral lipids in *Nannochloropsis oceanica* under nitrogen-depletion conditions. Algal Res..

[55] Li J, Han D, Wang D, Ning K, Jia J, Wei L (2014). Choreography of transcriptomes and lipidomes of *Nannochloropsis* reveals the mechanisms of oil synthesis in microalgae. Plant Cell.

[56] Taylor DC, Zhang Y, Kumar A, Francis T, Giblin EM, Barton DL (2009). Molecular modification of triacylglycerol accumulation by over-expression of *DGAT1* to produce canola with increased seed oil content under field conditions. Botany.

[57] Appleyard RK (1954). Segregation of new lysogenic types during growth of a doubly lysogenic strain derived from *Escherichia Coli* K12. Genetics.

[58] Elble R (1992). A simple and efficient procedure for transformation of yeasts. Biotechniques.

[59] Holsters M, de Waele D, Depicker A, Messens E, van Montagu M, Schell J (1978). Transfection and transformation of *Agrobacterium tumefaciens*. Mol Gen Genet.

[60] Clough SJ, Bent AF (1998). Floral dip: a simplified method for *Agrobacterium*-mediated transformation of *Arabidopsis thaliana*. Plant J.

[61] Deblock M, Debrouwer D, Tenning P (1989). Transformation of *Brassica napus* and *Brassica oleracea* using *Agrobacterium tumefaciens* and the expression of the *bar* and *neo* genes in the transgenic plants. Plant Physiol.

[62] Jefferson RA, Kavanagh TA, Bevan MW (1987). GUS fusions: β-glucuronidase as a sensitive and versatile gene fusion marker in higher plants. Embo J.

[63] Greenspan P, Mayer EP, Fowler SD (1985). Nile Red - a selective fluorescent stain for intracellular lipid droplets. J Cell Biol.

[64] Bligh EG, Dyer WJ (1959). A rapid method of total lipid extraction and purification. Can J Biochem Physiol.

[65] Katoh K, Toh H (2008). Recent developments in the MAFFT multiple sequence alignment program. Brief Bioinform.

[66] Price MN, Dehal PS, Arkin AP (2010). FastTree 2- approximately maximum-likelihood trees for large alignments. Plos One.

[67] Qi-Jun C, Hai-Meng Z, Jia C, Xue-Chen W (2006). Using a modified TA cloning method to create entry clones. Anal Biochem.

[68] An G (1985). High efficiency transformation of cultured tobacco cells. Plant Physiol.

[69] Genschik P, Criqui MC, Parmentier Y, Derevier A, Fleck J (1998). Cell cycle–dependent proteolysis in plants: identification of the destruction box pathway and metaphase arrest produced by the proteasome inhibitor MG132. Plant Cell.

