# Supplementary material for: Identification and characterization of an efficient acyl-CoA: diacylglycerol acyltransferase 1 (DGAT1) gene from the microalga Chlorella ellipsoidea
Source: BMC Plant Biol. 2017 Feb 21;17:48. doi: 10.1186/s12870-017-0995-5 (PMC5319178; doi:10.1186/s12870-017-0995-5)
Supplement: Additional file 1: Table S1. — Putative functional motifs in CeDGAT1 and AtDGAT1. (DOCX 17 kb) [file 12870_2017_995_MOESM1_ESM.docx]

**Table S1.** Putative functional motifs in CeDGAT1 and AtDGAT1.

| Functional site | CeDGAT1 | | AtDGAT1 | |  |
| --- | --- | --- | --- | --- | --- |
|  | Position Amino acid | | Position | Amino acid |  |
| *N*-Glycosylation | 160-163  373-376  467-470 | NQSV  NLSA  NLTL |  |  | |
| cAMP-/cGMP  dependent protein  kinase phosphorylation  [RK](2)-x-[ST]  Protein kinase C  phosphorylation  [ST]-x-[RK] | 83-86  229-232  335-338  419-422  8-10  59-61  108-110  125-127  228-230  251-253  469-471  490-492  491-493  524-526 | RKKS  KRHS  RKAS  RRLS  SQR  SQK  SEK  TKR  TKR  TER  TLR  TSK  SKR  TIK | 28-31  515-518  106-108  169-171  172-174  228-230  281-283 | RRKS  RKGS  TYR  SSR  SLR  TLR  SLK | |
| Casein kinase II  phosphorylation  [ST]-x(2)-[DE] | 92-95  106-109  108-111  162-165  177-180  184-187  188-191  590-593 | SVVD  SESE  SEKE  SVLD  TGLE  SLAD  TLPE  TIGD | 11-14  31-34  42-45  48-51  172-175  217-220  258-261  401-404 | TVTE  SRSD  SGSD  SPSD  SLRD  TMTE  TSYD  SVGD | |
| Tyrosine kinase phosphorylation  [RK]-x(2,3)-[DE] -x(2,3)-Y | 40-46 | KTWDLRY | 271-278  271-279 | KANPEVSY  KANPEVSYY | |
| *N*-Myristoylation G-  {EDRKHPFYW}-  x(2)-[STAGCN]-{P} | 170-175  178-183  212-217  690-695  708-713 | GSEESA  GLETAD  GMSESG  GQPMAI  GLANGS | 38-43  43-48  83-88  84-89  89-94  90-95  93-98  99-104  484-489  500-505 | GLLLSG  GSDNNS  GGGDNN  GGDNNG  GGGRGG  GGRGGG  GGGEGR  GNADAT  GSTVGN  GQPMCV | |
| Amidation x-G-[RK]-[RK]  Leucine zipper pattern L-x(6)-L-x(6)-L-x(6)-L | 81-84  458-461 | EGRK  PGKK | 222-243  229-250 | LYPVYVTLRCDS-AFLSGVTLML  LRCDSAFLSGVT-  LMLLTCIVWL | |
