# Supplementary material for: Identification and characterization of an efficient acyl-CoA: diacylglycerol acyltransferase 1 (DGAT1) gene from the microalga Chlorella ellipsoidea
Source: BMC Plant Biol. 2017 Feb 21;17:48. doi: 10.1186/s12870-017-0995-5 (PMC5319178; doi:10.1186/s12870-017-0995-5)
Supplement: Additional file 2: Figure S1. — RT-PCR detection of DGAT1 genes in transgenic yeast (INVSc1). The yeast actin was used as an internal control. 1, The yeast transformed with pYES2.0; 2–5, the yeast expressing AtDGAT1, GmDGAT1, BnDGAT1 and CeDGAT1, respectively. (DOCX 55 kb) [file 12870_2017_995_MOESM2_ESM.docx]

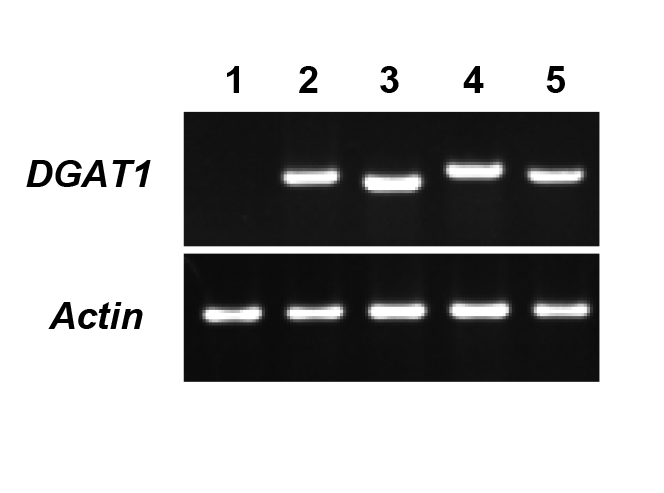


**Figure S1.** RT-PCR detection of *DGAT1* genes in transgenic yeast (INVSc1). The yeast *actin* was used as an internal control. 1, the yeast transformed with pYES2.0; 2-5, the yeast expressing *AtDGAT1*, *GmDGAT1*, *BnDGAT1* and *CeDGAT1*,respectively.
