# Supplementary material for: Identification and characterization of an efficient acyl-CoA: diacylglycerol acyltransferase 1 (DGAT1) gene from the microalga Chlorella ellipsoidea
Source: BMC Plant Biol. 2017 Feb 21;17:48. doi: 10.1186/s12870-017-0995-5 (PMC5319178; doi:10.1186/s12870-017-0995-5)
Supplement: Additional file 3: Figure S2. — A schematic map of the pCAMBIA2301-NOS-CeDGAT1-nos plasmid. The pCAMBIA2301-NOS-CeDGAT1-nos vector contained an expression box of the CeDGAT1 gene from C. ellipsoidea under the control of the NOS promoter and nos terminator; an expression box of the GUS gene controlled by the CaMV35S promoter and nos terminator; and an expression box of the kanamycin resistance gene, which conferred resistance to kanamycin. (DOCX 243 kb) [file 12870_2017_995_MOESM3_ESM.docx]

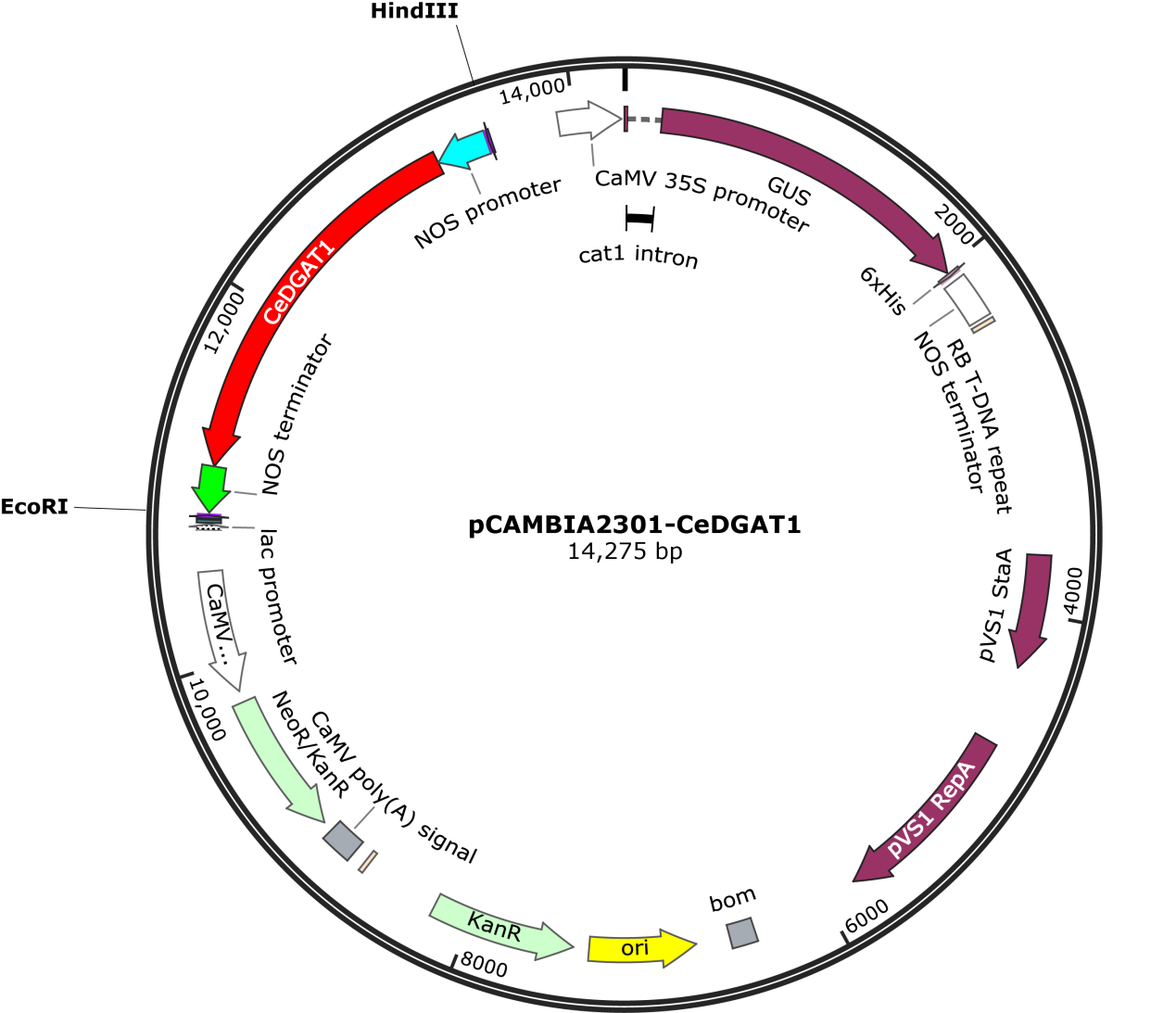


**Figure S2.** A schematic map of the pCAMBIA2301-NOS-CeDGAT1-nos plasmid. The pCAMBIA2301-NOS-CeDGAT1-nos vector contained: an expression box of the *CeDGAT1* gene from *Chlorella ellipsoidea* under the control of NOS promoter and nos terminator; an expression box of the *GUS* gene, controlled by the CaMV35S promoter and nos terminator; and an expression box of the kanamycin resistant gene, which conferred resistance to kanamycin.
