# Supplementary material for: Identification and characterization of an efficient acyl-CoA: diacylglycerol acyltransferase 1 (DGAT1) gene from the microalga Chlorella ellipsoidea
Source: BMC Plant Biol. 2017 Feb 21;17:48. doi: 10.1186/s12870-017-0995-5 (PMC5319178; doi:10.1186/s12870-017-0995-5)
Supplement: Additional file 4: Figure S3. — RT-PCR detection of CeDGAT1 in transgenic Arabidopsis (A) and B. napus (B) lines. Arabidopsis actin and B. napus GAPDH were used as internal controls. Col-0, wild-type Arabidopsis; 1, 9, and 12, transgenic Arabidopsis lines expressing NOS:CeDGAT1; Westar, wild-type B. napus var. Westar; 3, 10, 15, and 18, transgenic B. napus lines expressing NOS:CeDGAT1. (DOCX 93 kb) [file 12870_2017_995_MOESM4_ESM.docx]

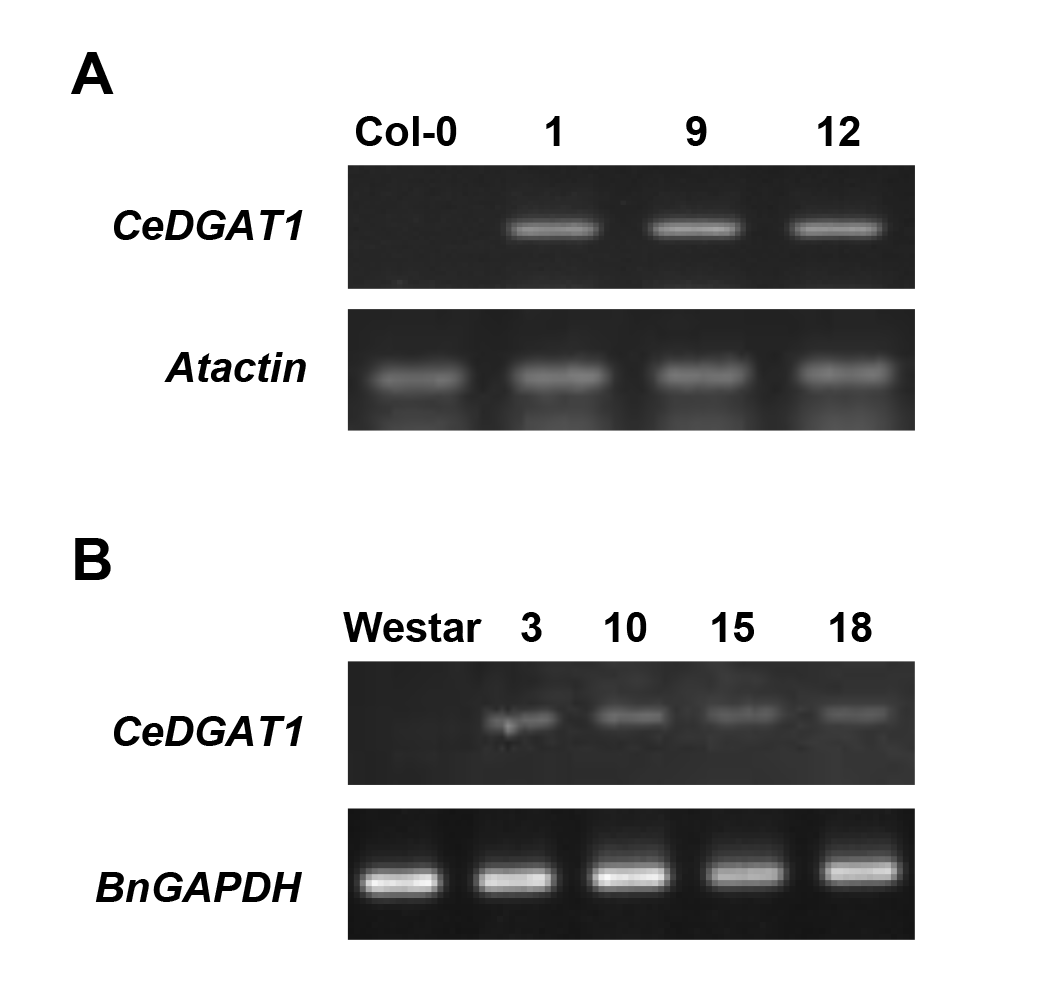


**Figure S3.** RT-PCR detection of *CeDGAT1* in transgenic Arabidopsis (A) and *B. napus* (B) lines. Arabidopsis *actin* and *B. napus GAPDH* were used as an internal control. Col-0, wild-type Arabidopsis; 1, 9, 12, transgenic Arabidopsis lines expressing NOS:CeDGAT1; Westar, wild-type *B. napus*; 3, 10, 15, 18, transgenic *B. napus* lines expressing NOS:CeDGAT1.
