# Supplementary material for: Identification and characterization of an efficient acyl-CoA: diacylglycerol acyltransferase 1 (DGAT1) gene from the microalga Chlorella ellipsoidea
Source: BMC Plant Biol. 2017 Feb 21;17:48. doi: 10.1186/s12870-017-0995-5 (PMC5319178; doi:10.1186/s12870-017-0995-5)
Supplement: Additional file 5: Figure S4. — PCR detection of the CeDGAT1 gene in transgenic B. napus lines. M, DNA molecular weight marker; 1, blank control; 2, wild-type B. napus var. Westar; 3–14, lines expressing NOS:CeDGAT1; 15, pCAMBIA2301-NOS-CeDGAT1-nos plasmid (positive control). (DOCX 112 kb) [file 12870_2017_995_MOESM5_ESM.docx]

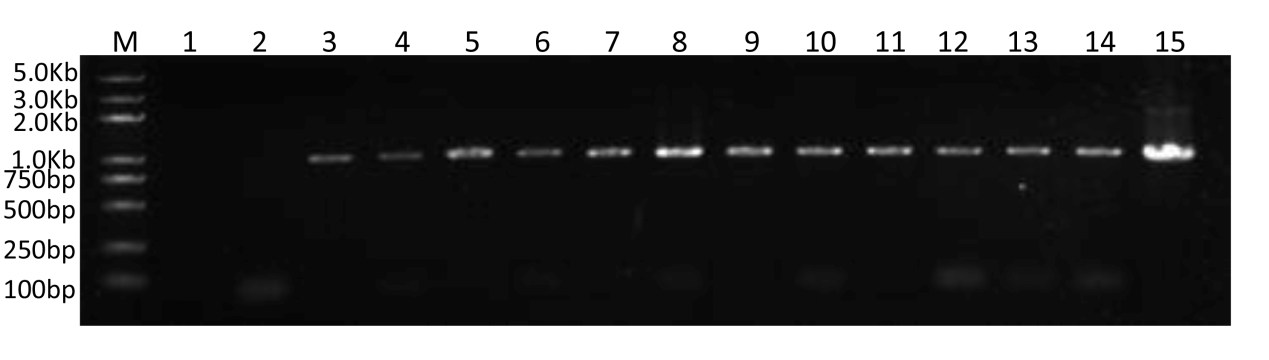


**Figure S4.** PCR detection of the *CeDGAT1* gene in transgenic *B. napus* lines. M, DNA molecular weight marker; 1, Blank control; 2, Wild-type *B. napus* var. Westar; 3-14, Lines expressing NOS:CeDGAT1; 15, pCAMBIA2301-NOS-CeDGAT1-nos plasmid (positive control).
