# Supplementary material for: Identification and characterization of an efficient acyl-CoA: diacylglycerol acyltransferase 1 (DGAT1) gene from the microalga Chlorella ellipsoidea
Source: BMC Plant Biol. 2017 Feb 21;17:48. doi: 10.1186/s12870-017-0995-5 (PMC5319178; doi:10.1186/s12870-017-0995-5)
Supplement: Additional file 6: Table S2. — List of primers. Sequences in lower-case letters indicate enzyme restriction sites. (DOCX 14 kb) [file 12870_2017_995_MOESM6_ESM.docx]

**Table S2.** List of primers.

| Experiment | Name of primers | Sequence (5’ to 3’) |
| --- | --- | --- |
| Amplifying *CeDGAT1* cDNA | P1 | ATGCCAGATGATGCCAGCA |
|  | P2 | TCAGCTGCCATTTGCGAG |
|  | P3 | CGCCaagcttATGCCAGATGATGCCAGCA |
|  | P4 | CCGGgaattcTCAGCTGCCATTTGCGAG |
| Amplifying *AtDGAT1* cDNA | P5 | CGCCaagcttATGGCGATTTTGGATTCTGC |
|  | P6 | CCGGgaattcTCATGACATCGATCCTTTTCG |
| Amplifying *GmDGAT1* cDNA | P7 | CGCCaagcttATGGCGATTTCCGATGAGCCTG |
|  | P8 | CCGGgaattcTCAGTCAAGTTTGCCTTTCCT |
| Amplifying *BoDGAT1* cDNA | P9 | CGCCaagcttATGGCGATTTTGGATTCTGG |
|  | P10 | CCGGgaattcTCAGGACATGGATCCTTTGC |
| RT-PCR of *CeDGAT1* | P11 | AGTCGGTTCTGGGTGTTCA |
|  | P12 | GCCTGAGTCGGAAGCATAGT |
| RT-PCR of *AtDGAT1* | P13 | TAACGATGACGCTCAGGGAA |
|  | P14 | AAAGGCAGCCAAAGGAAAGA |
| RT-PCR of *GmDGAT1* | P15 | TACCAGCCAAGCTATCCTCG |
|  | P16 | GGCATATTCCACATCCTCCA |
| RT-PCR of *BoDGAT1* | P17 | GGTTGATTCCGCTGTTGAGG |
|  | P18 | AAGGCAGCCAAAGGAAAGAT |
| RT-PCR of yeast *actin* | P19 | ACGTCGCCTTGGACTTCGAA |
|  | P20 | AGATGGAGCCAAAGCGGTGA |
| Transformants confirmation of *CeDGAT1* | P21 | AACTGACAGAACCGCAACG |
|  | P22 | CCTGAGTCGGAAGCATAGTC |
| RT-PCR of Arabidopsis *actin* | P23 | ATGACATGGAGAAGATCTGGCATCA |
|  | P24 | AGCCTGGATGGCAACATACATAGC |
| RT-PCR of *B. napus GAPDH* | P25 | TCAAGAAGGCTATCAAGGAG |
|  | P26 | GTAACCCCATTCGTTGTCAT |
| qRT-PCR detection of *CeDGAT1* | P27 | AGTCGGTTCTGGGTGTTCA |
|  | P28 | ACATCCCGCCTCTACTAAGG |
| 18S rRNA | P29 | CTTGTAAACCGCGTCGTGATG |
|  | P30 | GACGTAATCAACGCGAGCTGAT |

Sequences in lower-case letters indicate enzyme restriction sites.
