# Supplementary figures and images for: Identification and characterization of an efficient acyl-CoA: diacylglycerol acyltransferase 1 (DGAT1) gene from the microalga Chlorella ellipsoidea
Source: BMC Plant Biol. 2017 Feb 21;17:48. doi: 10.1186/s12870-017-0995-5 (PMC5319178; doi:10.1186/s12870-017-0995-5)

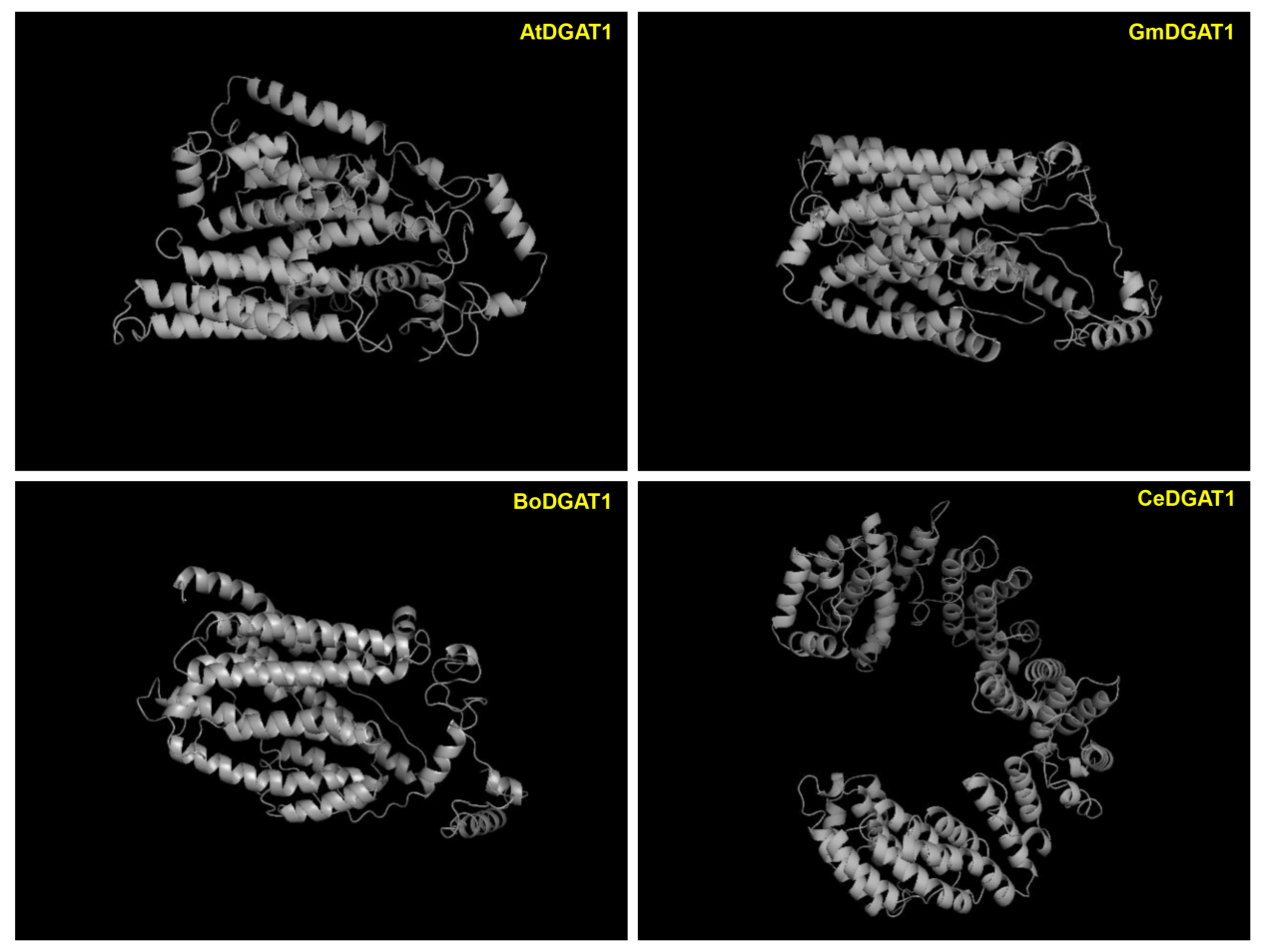


**Figure S5.** Comparison of the predicted structurs of CeDGAT1, AtDGAT1, GmDGAT1 and BoDGAT1.

Supplement: Additional file 7: Figure S5. — Comparison of the predicted structures of CeDGAT1, AtDGAT1, GmDGAT1 and BoDGAT1. (DOCX 416 kb) [file 12870_2017_995_MOESM7_ESM.docx]
